# Elucidating the role of *AC026412.3* in hepatocellular carcinoma: a prognostic disulfidptosis-related LncRNAs model perspective

**DOI:** 10.1186/s12876-025-04174-6

**Published:** 2025-08-12

**Authors:** Qinghua Ji, Chuanbing Shi, Xuefeng Gu, Ling Yang, Yintao Sun

**Affiliations:** 1 Department of Gastroenterology, Zhongda Hospital Affiliated to Southeast University, Nanjing, Jiangsu China; 2 Department of Pathology, Nanjing Pukou People’s Hospital, Liangjiang Hospital, Southeast University, Nanjing, Jiangsu China; 3 Department of Central Laboratory, Jurong Hospital Affiliated to Jiangsu University, 66 Ersheng Road, Zhenjiang, Jiangsu China; 4 Department of Imaging, the Second People’s Hospital of Changzhou, the Third Affiliated of Nanjing Medical University, 68 Gehu Road, Wujin District, Changzhou, Jiangsu China

**Keywords:** Hepatocellular carcinoma, Disulfidptosis, Long noncoding RNA, Bioinformatics, Invasion, Migration, Metastasis, *AC026412.3*

## Abstract

Disulfidptosis—a newly characterised mode of regulated cell death implicated in tumorigenesis—exhibits undefined prognostic utility in hepatocellular carcinoma (HCC), particularly concerning disulfidptosis-related long non-coding RNAs (DRLs). Integrating transcriptomic and clinical data from The Cancer Genome Atlas, we identified 807 DRLs and constructed a prognostic signature via univariate Cox regression, LASSO-Cox penalisation, and multivariate Cox analysis. The resulting four-DRL signature (*AL031985.3*,* TMCC1-AS1*,* AL590705.3*,* AC026412.3*) stratified patients into distinct risk cohorts, with high-risk groups demonstrating significantly reduced overall survival (OS; log-rank *P* < 0.001) and hazard ratios independent of conventional clinicopathological variables. Model discrimination was robust across multiple metrics: time-dependent receiver operating characteristic curves yielded AUCs of 0.750 (95% CI: 0.676–0.817) at 1 year, 0.709 (0.637–0.781) at 3 years, and 0.720 (0.641–0.799) at 5 years, outperforming established staging systems. Concordance indices (C-index: 0.681) and principal component analysis further validated stratification efficacy. Functional annotation linked the signature to extracellular matrix dysregulation, epithelial-mesenchymal transition, and immunosuppressive microenvironments. High-risk patients exhibited elevated tumour mutational burden (*P* = 0.04), increased M0 macrophage infiltration, and heightened tumour immune dysfunction and exclusion (TIDE) scores (*P* < 0.001)—indicating impaired immunotherapy response. Pharmacogenomic profiling revealed enhanced sensitivity to five agents in high-risk subgroups (BDP-00009066, GDC0810, Osimertinib, Paclitaxel, YK-4-279; all *P* < 0.01). Critical experimental validation confirmed *AC026412.3* as an oncogenic driver: significantly overexpressed in HCC tissues (*P* < 0.001) and cell lines, its knockdown suppressed proliferation, invasion, and migration in vitro. In vivo models demonstrated its necessity for angiogenesis (chorioallantoic membrane assay), primary tumour growth (orthotopic implantation), pulmonary metastasis, and epithelial-mesenchymal transition activation. This molecularly annotated signature enables precise prognostic stratification and guides personalised therapeutic strategies in HCC.

**Supplementary Information:**

The online version contains supplementary material available at 10.1186/s12876-025-04174-6.

## Introduction

Hepatocellular carcinoma (HCC), the predominant form of primary liver cancer, represents a formidable global health challenge. It ranks as the sixth most commonly diagnosed cancer and the third leading cause of cancer-related mortality worldwide, accounting for approximately 8.3% of all cancer deaths [1, 2]. Current projections estimate the incidence of liver cancer will approach one million cases annually by 2025 [3]. Major aetiologies include chronic hepatitis B (HBV) and C virus infections, non-alcoholic fatty liver disease (NAFLD), and alcohol-related liver disease [4, 5]. While therapeutic options encompass surgical resection, ablation, locoregional therapies (transarterial chemoembolization, radioembolization), and systemic agents (including molecular-targeted therapies and immune checkpoint inhibitors) [6–9], the prognosis remains dismal, with a 5-year relative survival rate of only 20% [2]. This underscores the critical need for improved prognostic stratification tools and novel therapeutic targets.

Prognostic assessment in HCC has traditionally relied on clinicopathological staging systems such as the Barcelona Clinic Liver Cancer (BCLC), Cancer of the Liver Italian Program (CLIP), and Tumor-Node-Metastasis (TNM) classifications [10]. While valuable, these systems exhibit limitations in accuracy and fail to fully capture the underlying molecular heterogeneity driving tumour behaviour and treatment response [10, 11]. Consequently, significant research efforts focus on identifying molecular biomarkers to refine prognostication. Within this landscape, long non-coding RNAs (lncRNAs) – transcripts longer than 200 nucleotides with limited protein-coding potential – have emerged as potent regulators of carcinogenesis, influencing key processes like proliferation, metastasis, apoptosis evasion, and treatment resistance [12, 13]. Their expression profiles demonstrate considerable prognostic value across various cancers, including HCC [14–16]. Numerous lncRNA-based prognostic signatures have been developed, integrating factors related to angiogenesis, exosomes, immune responses, glycolysis, and various forms of regulated cell death (RCD) [17–23]. However, many existing models require further external validation and lack integration of the most recently discovered RCD pathways [11, 24].

Regulated cell death RCD mechanisms are fundamental to maintaining cellular homeostasis, and their dysregulation is a recognized hallmark of cancer, enabling tumour cells to evade destruction [25, 26]. Beyond the well-established apoptosis, emerging forms of RCD, such as ferroptosis, necroptosis, pyroptosis, and cuproptosis, offer novel insights into cancer biology and therapeutic opportunities [27, 28]. Disulfidptosis represents a recently elucidated form of RCD, distinct from known mechanisms [29]. It occurs in cells with high *SLC7A11* expression under glucose deprivation, where aberrant disulfide bond formation disrupts the actin cytoskeleton, leading to catastrophic cell death [29]. This unique mechanism, involving *SLC7A11*-mediated cystine uptake, NADPH-dependent reduction, and thioredoxin system regulation [30], presents a promising vulnerability for therapeutic targeting [29]. Recent mechanistic advances highlight the sophisticated regulatory networks governing disulfidptosis beyond canonical pathways. Epigenetic modulation by histone methyltransferase SETD1B, stabilised via USP15-mediated deubiquitination, drives disulfidptosis in neuronal cells by enhancing H3K4me3 enrichment at promoters of cytoskeletal regulators NCKAP1L and WASF2 (WAVE-2), thereby amplifying actin disulfide crosslinking during ischemic stress [31]. In HCC, mitochondrial dysfunction constitutes a pivotal initiator: Alexidine dihydrochloride (AD) triggers mitochondrial ultrastructural damage and reactive oxygen species (ROS) overproduction, activating the ATF4-DDIT3 axis to escalate disulfide stress and potentiate disulfidptosis [32]. Conversely, dipeptidyl peptidase 7 (DPP7) promotes colorectal cancer progression by binding glutathione peroxidase 4 (GPX4), stabilising this redox guardian to suppress cytoskeletal disulfide aggregation (e.g., in drebrin, FLNA/B) and evade NK cell cytotoxicity [33]. In the tumour microenvironment, lactate dehydrogenase B (LDHB) critically regulates disulfidptosis in CD8⁺ T cells: *SLC7A11*-mediated cystine uptake induces disulfide stress, while LDHB interaction with G6PD depletes NADPH and drives disulfidptosis, leading to T cell exhaustion and compromised antitumour immunity via STAT3-dependent LDHB overexpression [34].These findings underscore organ-specific vulnerability to disulfidptosis, modulated by ubiquitination, organelle stress, and immune-editing pathways.

Although lncRNA prognostic models constructed based on disulfidptosis have been associated with other gastrointestinal malignancies such as esophageal squamous cell carcinoma [35, 36], and some studies have even conducted external validation of these models [37], the specific roles of disulfidptosis-related lncRNAs (DRLs) in the onset, progression, and prognosis of HCC remain inadequately explored. Programmed cell death has consistently been a central focus of our research endeavors. Previously, we developed a prognostic model for hepatocellular carcinoma HCC based on cuproptosis-related lncRNAs [38]. Recently, our research attention has shifted towards elucidating the role and significance of disulfidptosis-related lncRNAs in HCC [39, 40]. To further investigate the implications and functions of these DRLs in HCC, we have undertaken the present study. Given the critical role of lncRNAs in modulating cellular pathways, including RCD [12, 13], and the emerging significance of disulfidptosis in cancer [29, 30], we hypothesize that DRLs are crucial players in HCC biology. Integrating DRLs into prognostic models offers the potential to significantly enhance risk stratification beyond conventional staging and existing lncRNA signatures. Among DRLs identified through preliminary bioinformatic analysis of The Cancer Genome Atlas (TCGA) HCC data, *AC026412.3* emerged as a compelling candidate. It exhibits significant overexpression across major HCC aetiological subgroups compared to paracancerous tissue and lacks prior functional characterization or mechanistic insight regarding its role in HCC or disulfidptosis. Therefore, this study aims to: (1) Construct and validate a novel prognostic signature based on DRLs for HCC patients; (2) Comprehensively analyse the association of this signature with the tumour immune microenvironment, immunotherapy response potential, tumour mutational burden (TMB), and chemotherapeutic drug sensitivity; and (3) Experimentally validate the expression and functional impact of key signature DRLs, with a specific focus on elucidating the oncogenic role and potential mechanism of action of *AC026412.3* in HCC progression and metastasis.

## Materials and methods

### HCC dataset and preprocessing

Transcriptome data for HCC analysis were obtained from the TCGA database (https://portal.gdc.cancer.gov/). After excluding samples with survival time of 0 days or incomplete survival data, 365 HCC patients were included in the analysis. Raw RNA-seq counts (HTSeq-Counts) were preprocessed. Compositional biases were corrected using the Trimmed Mean of M-values (TMM) method in edgeR (v4.0.0). Low-expression genes were filtered by retaining those with counts per million (CPM) > 1 in at least 50% of samples. Expression values for genes with multiple Ensembl identifiers were averaged to generate a single value per gene. Data were then log₂(CPM + 1) transformed. Principal component analysis (PCA) revealed no significant technical batch effects, so batch correction was omitted. The median follow-up duration was 15.6 months (range: 7.1–28.5 months). OS was defined as the time from initial pathological diagnosis to death from any cause; patients alive at last follow-up were right-censored. Progression-free survival (PFS) was defined as the time from diagnosis to recurrence, progression, or death from any cause. Additional clinical data were obtained from the cBioPortal database (http://www.cbioportal.org/).

We benchmarked the prognostic performance of the riskScore model against established clinical scoring systems (BCLC Staging, ALBI Score, CLIP Score, AFP scoring system) using curated TCGA-LIHC data. As these systems were not natively annotated in TCGA, we implemented validated mapping protocols. BCLC staging was reconstructed according to AASLD guidelines by integrating AJCC-TNM (8th edition), Child-Pugh grade, and ECOG performance status. Metastatic status was prioritized: M1 patients were classified as Stage C; M0/MX patients were non-metastatic. Non-metastatic patients were staged as: Stage A (T1), Stage B (T2), Stage C (T3/T4, N1, or ECOG 1–2), or Stage D (Child-Pugh C or ECOG > 2). For the ALBI score, units for serum albumin and total bilirubin were standardized: albumin values with normal range lower bounds < 10 g/dL were converted to g/L (×10), and bilirubin (mg/dL) was converted to µmol/L (× 17.1). The ALBI score was calculated as (log₁₀[bilirubin] × 0.66) - (albumin × 0.085) for grade stratification. The CLIP score (0–6) was derived from Child-Pugh points (A = 0, B = 1, C = 2), tumour morphology (approximated by AJCC T-stage: T1 = 0, T2 = 1, T3/T4 = 2), AFP thresholds (< 400 = 0, 400–1000 = 1, > 1000 ng/mL = 2), and portal thrombosis (inferred from macroscopic vascular invasion = 2). The AFP scoring system used the same thresholds as the CLIP AFP component. Missing data were imputed using the “rpart” package in R.

Treatment heterogeneity was addressed by classifying patients based on clinical records using a hierarchical protocol: (1) ‘Adjuvant Therapy’ (radiation or pharmaceutical adjuvant, including chemotherapy), (2) ‘Ablation/Embolization’, (3) ‘Surgery Only’ (definitive surgery without adjuvant), (4) ‘Other/Unknown’ (supportive care or incomplete records). Patients receiving both surgery and adjuvant therapy were classified as ‘Adjuvant Therapy’ to reflect the potential prognostic impact of multimodal treatment. This classification enabled stratified evaluation of the lncRNA signature’s prognostic performance across therapeutic contexts.

To address potential heterogeneity in treatment approaches among HCC patients within the TCGA cohort, we implemented a comprehensive treatment classification system based on clinical intervention records. Treatment modalities were categorised according to a hierarchical protocol: (1) Patients receiving either radiation therapy or pharmaceutical adjuvant treatment (including chemotherapy) were classified under the ‘Adjuvant Therapy’ category. (2) Those undergoing ablation or embolization procedures were designated to the ‘Ablation/Embolization’ group. (3) Individuals undergoing definitive surgical procedures (e.g., lobectomy, segmentectomy, or extended resection) without documented adjuvant therapies were classified as ‘Surgery Only’. (4) All remaining cases, including those with supportive care only or incomplete treatment documentation, were categorised as ‘Other/Unknown’. Notably, patients receiving combined surgical and adjuvant interventions were prioritised under adjuvant categories, reflecting the potential prognostic influence of multimodal therapy. This classification schema enabled stratified evaluation of our lncRNA signature’s prognostic performance across distinct therapeutic contexts.

Perl (version Strawberry-5.30.1; https://www.perl.org) was used to identify 16,876 lncRNAs and 19,938 mRNAs expressed in HCC samples. Ten disulfidptosis-related genes (*OXSM*,* GYS1*,* NUBPL*,* NDUFS1*,* LRPPRC*,* NCKAP1*,* NDUFA11*,* SLC3A2*,* RPN1*,* SLC7A11*) were derived from previous studies [citation needed]. Their expression and prognostic value in HCC were assessed. DRLs were identified by assessing the correlation between the expression of these ten genes and all lncRNAs using Wilcoxon rank-sum tests (R function wilcox.test()). Significant correlations were defined as |correlation coefficient| > 0.4 with FDR < 0.01. LncRNAs meeting these criteria were designated as DRLs. A Sankey diagram illustrating gene co-expression was generated using ggplot2 (v3.5.0) and ggalluvial (v0.12.5) R packages. Patient data were obtained from TCGA under its publication guidelines; therefore, specific ethical approval for this secondary analysis was waived.

### Development and verification of a DRLs-based prognostic signature

The TCGA-LIHC cohort was randomly divided into training (*n* = 183) and test (*n* = 182) sets. Using the training cohort, we first performed univariate Cox regression (*P* < 0.05) to identify DRLs significantly associated with OS. To mitigate overfitting, LASSO Cox regression [41] was applied using the glmnet package (v4.1-8). Ten-fold cross-validation was performed (maximum iterations = 1000) to identify prognostic DRLs. The optimal lambda (lambda.min), minimizing cross-validation error, was selected using the cv.glmnet function. Significant DRLs from LASSO underwent multivariate Cox regression. The final prognostic model was selected using stepwise regression (both directions) based on the Akaike Information Criterion (AIC) via the R step function. The model was validated in the test cohort and the entire TCGA-LIHC dataset. (Detailed protocols are in Supplementary Methods). Patients in the training cohort were stratified into high- and low-risk groups based on the median risk score. The risk score for each patient was calculated as the sum of each included lncRNA’s expression multiplied by its Cox regression coefficient. Kaplan-Meier (KM) survival analysis [42] (Survival v3.8-3, Survminer v0.5.0) compared survival between risk groups (log-rank test p-value). Time-dependent ROC curves evaluated prognostic performance at 1, 3, and 5 years. Principal component analysis (PCA) using limma (v3.58.1) and visualization with scatterplot3d (v0.3.44) assessed distribution separation between risk groups. Univariate and multivariate Cox regression analyses assessed whether the risk score was an independent prognostic factor. A nomogram integrating the risk score and clinical factors to predict 1-, 3-, and 5-year survival was constructed using the rms package (v6.8.0). Calibration curves assessed the nomogram’s predictive accuracy.

### Gene Ontology (GO), Gene Set Enrichment Analysis (GSEA), and Protein-Protein Interaction (PPI) networks analysis

We adopted the R limma (v3.58.1) package to identify differentially expressed genes (DEGs) between the low- and high-risk groups, with screening criteria set as log2 |fold change| > 2, false discovery rate (FDR) < 0.01. GO analysis of DEGs was on the low and high-risk groups; GO pathways consist of three components: biological process (BP), molecular function (MF), and cell component (CC). *P* < 0.05 and FDR < 0.01 denoted biological processes and pathways that exhibited substantial enrichment. We then performed GSEA [43] to elucidate primary signaling pathways and molecular functions. The gene sets used for GSEA were c2.all.v2022.1.Hs.symbols.gmt, c5.all.v2022.1.Hs.symbols.gmt, and h.all.v2022.1.Hs.symbols.gmt. All enrichment analyses were based on R packages org.Hs.eg.db (v3.18.0), clusterProfiler (v4.10.1) [44], and enrichment plot. Furthermore, DEGs were input into the STRING database (https://string-db.org/) to establish the initial network (confidence score set as ≥ 0.7) [45]. In this network, genes without interactions were eliminated; the PPI’s hub genes in the network were defined as genes with connection numbers ≥ 5.

### Tumor immune microenvironment, immunotherapy, and TMB analysis

Immune cell infiltration was quantified using CIBERSORT [46], obtaining the abundance of immune cells in every sample of HCC patients, and the results were screened by *P* < 0.05. The R packages, which comprised of reshape2 (v1.4.4), limma (v3.58.1), and ggpubr (v0.6.0) analyzed the variations in individual immune cell abundance between the low- and high-risk groups and thereafter plotted the box plots. Single-sample gene set enrichment analysis (ssGSEA) [47] assessed differences in immune function between the two risk groups to evaluate the differences in the HCC tumor immune microenvironment between them, using the R packages reshape2 (v1.4.4), limma (v3.58.1), GSEABase (v1.64.0), and GSVA (v1.50.5).

Next, based on simulating mechanisms of tumor immune escape, the tumor immune dysfunction and exclusion (TIDE) algorithm [48] executed the prediction of the immune treatment responses, observing the effects of immune treatment between low- as well as the high-risk groups. The mutation data of the downloaded HCC samples from TCGA were checked and integrated with the R package “maftools (v2.18.0)” [49], and TMB waterfall plots of the top 15 genes that composed the highest mutation frequency in TCGA-LIHC were plotted for the low as well as the high-risk groups correspondingly. Finally, TMB difference analysis was performed between the low and high-risk groups, and TMB survival curves and their integration with the risk score were plotted.

### OncoPredict applied for drug sensitivity analysis

Drug sensitivity was predicted using the oncoPredict R package (v1.2) [50]. The drug sensitivity of every sample was first scored utilizing the R package oncoPredict, predicting the half maximal inhibitory concentration (IC50) values of HCC samples for various anti-tumor drugs. Then, the R packages ggpubr (v0.6.0) and limma (v3.58.1) were adopted to compare drug sensitivities between low and high-risk groups to identify drug resistance and sensitivity, where *P* < 0.05 denoted the significant threshold.

### Bioinformatics analysis of prognostic drls in the model

We performed bioinformatics analysis on the prognostic DRLs in the risk model.

First, patients were stratified by median expression of each prognostic DRL, and KM survival analysis was performed. Differential expression of each prognostic DRL across 33 TCGA cancer types was analyzed using Wilcoxon tests (plyr v1.8.9, ggpubr v0.6.0, reshape2 v1.4.4). Pearson correlation analysis was conducted to derive mRNA genes that were substantially correlated with prognostic DRLs, with the filtering criteria for the correlation coefficient set at 0.4 and the filtering criteria for the correlation test p-value set at *p* < 0.001. The findings were visualized using the R package ComplexHeatmap (v2.18.0). GO analysis was performed on co-expressed mRNAs. GSEA was performed based on prognostic DRL expression groups. Drug sensitivity and immune cell infiltration analyses were also performed for each prognostic DRL. Finally, the expression differences, ROC, and time-dependent ROC of the prognostic DRLs were further analyzed to identify the diagnostic value of the risk prognostic DRLs for clinical treatment. The association of three hub DRLs’ expression with clinical/pathological features was assessed using ggplot2 (v3.5.0) and reshape2 (v1.4.4).

### Cell culture and transfection

Human HCC cell lines and the normal hepatic LO2 cell line were obtained from the Cell Bank of the Chinese Academy of Sciences (Shanghai, China). Ethical approval was obtained from the Ethics Committee of Jurong Hospital, affiliated with Jiangsu University. Cells were maintained in Dulbecco’s Modified Eagle Medium (DMEM; Gibco) supplemented with 10% fetal bovine serum (FBS; Gibco) to support growth. Cultures were incubated under humidified conditions at 37 °C with 5% CO₂. LncRNA *AC026412.3* expression was knocked down in HCC cells using specific siRNAs (GenePharma, China). Transfection of HCC cells was performed using Lipofectamine™ 3000 (Invitrogen, USA).

### Knockdown efficiency was confirmed by qRT-PCR

*AC026412.3* expression was validated in clinical HCC samples (*n* = 20 paired tumor/adjacent tissues) and HCC cell lines (HepG2, Hep3B2.1-7, HCC-LM3) versus the normal LO2 cell line. Twenty cases of fresh tissue samples of HCC tumors and adjacent cancer were obtained from Jiangsu Provincial Cancer Hospital. Specimens were selected based on the following criteria: (i) histopathological confirmation of HCC diagnosis; (ii) availability of matched fresh-frozen tumour and adjacent normal tissue (> 2 cm from tumour margin); (iii) no prior neoadjuvant therapy (chemotherapy or radiotherapy). A power analysis (Supplementary Fig. 1) was performed for the paired clinical samples (*n* = 20) using the pwr package in R. This analysis, assuming an α-level of 0.05, indicated a large effect size (Cohen’s d = 0.805) and a statistical power of 92.7%, substantially exceeding the conventional 80% threshold. The analysis further determined that a minimum sample size of 15 paired specimens would be required to achieve 80% power, confirming the adequacy of the 20 paired samples for robust detection of the observed effects. Total RNA was extracted from all samples using TRIzol reagent (Invitrogen, Thermo Fisher Scientific, Carlsbad, CA, USA) strictly according to the manufacturer’s protocol. RNA concentration and purity were quantified using a NanoDrop 2000 spectrophotometer (Thermo Fisher Scientific, Waltham, MA, USA). Subsequently, 1 µg of total RNA from each sample was reverse transcribed into complementary DNA (cDNA) using the HiScript 1 st Strand cDNA Synthesis Kit (Vazyme, China). QRT-PCR was performed using the synthesized cDNA as template and the SYBR Green Kit (Vazyme, China) for amplification, following standard protocols. GAPDH served as the endogenous control. All reactions were run in technical triplicates. Relative quantification of *AC026412.3* expression levels was determined using the comparative 2^(−ΔΔCt) method, with GAPDH expression providing the normalization baseline. The specific primer sequences employed were: the forward primer for *AC026412.3* is CTCTAGTGAGCGGGTGTGAC and the reverse primer is GAGCCAAAGGGATCTACGCA. For GAPDH, the forward primer is GCACCGTCAAGGCTGAGAAC, while the reverse primer is TGGTGAAGACGCCAGTGGA.

### Cell proliferation assay

Cellular proliferation was evaluated using the Cell Counting Kit-8 (CCK-8, Beyotime Biotechnology, China). Briefly, HCC cells were plated into 96-well flat-bottom microplates (Corning Inc., USA) at an initial density of 2 × 10³ cells per well in 100 µL complete medium. Following overnight incubation (37 °C, 5% CO₂ humidified atmosphere), cells were treated according to experimental protocols. At designated time points (24, 48, 72, and 96 h post-treatment), cell viability was quantified. The culture medium in each well was replaced with 100 µL of serum-free DMEM (Gibco, Thermo Fisher Scientific, USA) containing 10% (v/v) CCK-8 reagent. Plates were then incubated for 2 h at 37 °C. Finally, the absorbance of the formazan product was measured at 450 nm using a microplate reader (SpectraMax M3, Molecular Devices, USA). Relative cell proliferation was calculated based on optical density (OD) values.

To assess colony-forming capacity, HCC cells were seeded into 6-well plates at a low density of 500 cells per well. Cells were cultured under standard conditions (37 °C, 5% CO₂) for approximately 14 days, allowing macroscopic colony formation. Upon colony development, the culture medium was aspirated. Cells were fixed by incubating with 4% paraformaldehyde for 20 min at room temperature. After fixation, the solution was removed, and the wells were gently rinsed twice with phosphate-buffered saline (PBS). Subsequently, colonies were stained with a 0.5% (w/v) crystal violet solution for 20 min. Excess stain was thoroughly removed by washing with distilled water. The stained colonies were air-dried, photographed digitally, and manually counted. Colonies exceeding approximately 50 cells or 50 μm in diameter were considered for counting.

### Wound healing aassay

Following cell quantification, HCC-LM3 cells from each experimental group were plated into six-well plates at an initial density of approximately 6 × 10⁵ cells per well in complete medium. Cells were allowed to adhere and form a confluent monolayer during a 12-hour incubation period at 37 °C under 5% CO₂. To create a uniform cell-free region, a linear scratch wound was meticulously generated across the monolayer using a sterile 200-µL pipette tip. Subsequent to wounding, each well was gently rinsed twice with PBS to remove dislodged cellular debris. Fresh serum-free basal medium was then added to the wells to minimize the confounding effects of cell proliferation on migration. The initial wound area (t = 0 h) was immediately documented using phase-contrast microscopy. Migration into the denuded area was monitored, and images of the identical wound region were captured again after a 48-hour incubation period. Quantitative assessment of the wound closure rate was performed by measuring the residual wound area at both time points utilizing ImageJ software.

### Transwell migration and invasion

To evaluate migratory and invasive capabilities, HCC cells from experimental groups were harvested, suspended in serum-free DMEM (Gibco, Thermo Fisher Scientific, Waltham, MA, USA), and adjusted to a density of 2 × 10⁵ cells/mL. Cell suspensions (200 µL) were seeded into the upper chambers of transwell inserts (8 μm pore size; Corning Inc., Corning, NY, USA) placed within 24-well plates. For invasion assays specifically, inserts were pre-coated with a layer of Matrigel matrix (diluted to 20% in serum-free medium; Corning Matrigel Matrix, Corning Inc.) to simulate the extracellular matrix barrier. The lower chambers were filled with 600 µL of DMEM supplemented with 10% fetal bovine serum (FBS; Gibco) serving as a chemoattractant. Cells were incubated under standard conditions (37 °C, 5% CO₂ humidified atmosphere) for 24 h. Following incubation, non-migrated/non-invaded cells residing on the upper membrane surface were removed using sterile cotton swabs (Puritan Medical Products, Guilford, ME, USA). Cells that had traversed the membrane were fixed to the lower surface with 4% paraformaldehyde (Sigma-Aldrich, St. Louis, MO, USA) for 15 min and subsequently counterstained with 0.4% crystal violet (Sigma-Aldrich) for 15 min. Representative microscopic fields of the stained cells were captured using an inverted microscope, and the number of migrated or invaded cells was quantified by counting.

### CAM assay in chick embryos

Fresh fertilised eggs from White Laihang chickens were obtained from Shanghai Huguang Poultry Breeding Co., Ltd. These eggs underwent surface sterilisation using 70% ethanol before being incubated under controlled conditions of 37 °C and 65% humidity, commencing from the first day of embryo development (EDD-1). On the third day of incubation (EDD-3), a small opening was made at the apex of each egg. This opening was sealed with cellophane to maintain appropriate moisture levels and to prevent contamination. Subsequently, the eggs were incubated in an upright, stationary position until EDD-10. At this stage (EDD-10), the viability of the embryos and the vascular network of the CAM were assessed visually before further experimental manipulation. All procedures involving the embryos adhered to ethical guidelines, ensuring termination did not exceed EDD-17. For the experimental setup, stably transfected HCC-LM3 cells, either with sh-*AC026412.3* or a non-specific short hairpin RNA control (sh-NC), were suspended in a 1:1 ratio with growth factor-reduced Matrigel. This cell-Matrigel mixture, containing 5 × 10^5 cells per embryo, was applied carefully to the CAM’s surface. Post-implantation, the apertures were resealed with tape, and the eggs were returned to the incubator. Starting from the day of transplantation, observations of the eggs were conducted daily until EDD-14. On EDD-14, vascular growth was examined macroscopically using a stereomicroscope. The extent of angiogenesis was quantified by enumerating the vascular branches present within each sample.

### Establishment of an orthotopic hepatocellular carcinoma model using HCCLM3 cells in nude mice

BALB/c nude mice of specific pathogen-free (SPF) grade (Shanghai SLAC Laboratory Animal Co. Ltd) were used to generate an orthotopic HCC model. Mice were randomly allocated to control or experimental groups using a computer-generated sequence to ensure unbiased group assignment and baseline comparability. Orthotopic xenograft studies utilised six male BALB/c nude mice per experimental group. Mice were allocated into two groups and inoculated with 1 × 10^6 stably transfected HCC-LM3 cells (sh-*AC026412.3* or sh-NC) directly into the liver lobe capsule. Eight weeks post-inoculation, mice were euthanized, and liver tissues were collected and stored in liquid nitrogen for future analyses. To ensure the highest standards of animal welfare by minimizing pain and stress, a combined methodology of anesthesia and euthanasia was employed. The mice were euthanized via cervical dislocation following anesthesia with an intraperitoneal injection of sodium pentobarbital at a dose of 50 mg/kg. The animal welfare and experimental protocols were approved by the Medical Ethics Committee of Jurong Hospital Affiliated to Jiangsu University. All experiments were conducted in accordance with the Guide for the Care and Use of Laboratory Animals. Anaesthesia (intraperitoneal sodium pentobarbital, 50 mg/kg) and humane endpoints were employed to minimise distress. Euthanasia was performed by cervical dislocation under deep anaesthesia.

### Establishment of a mouse model for lung metastasis

Male athymic nude mice, aged six weeks, were sourced from Shanghai SLAC Laboratory Animal Co. Ltd, China. The mice were maintained under standard laboratory conditions at our institution’s animal care facility. The mice were randomly divided into two groups using computer-generated sequences: the sh-*AC026412.3* interference group and the NC group. To induce lung metastasis, LM3 HCC cells, either with pL-NC or pL-sh-*AC026412.3* transfection, were suspended in 200 µl of PBS and injected intravenously via the tail vein. Each experimental group comprised five mice. The efficacy of sh-*AC026412.3* interference was validated by qRT-PCR prior to cell administration. After an eight-week period, the mice were humanely sacrificed for further investigation. Metastatic lung involvement was visualised through photographic analysis, and the presence of HCC cells was assessed using haematoxylin and eosin (H&E) staining. QRT-PCR was employed to measure the expression levels of E-cadherin, Vimentin, matrix metalloproteinase 9 (MMP9), and Ki-67 in the lung tissue, with immunohistochemistry (IHC) serving as an additional verification method. All experimental protocols were conducted in triplicate and were repeated on multiple occasions to ensure data reliability and reproducibility.

### IHC

To investigate the impact of *AC026412.3* on the proliferation, invasion, and metastasis of HCC cells, as well as its association with EMT, mouse-derived tissue samples were subjected to fixation in 4% paraformaldehyde for 48 h. Thereafter, the tissues were sectioned into slices of 5 μm thickness. To prevent nonspecific binding, the sections were initially treated with 5% goat serum. Subsequently, they were incubated with the following primary antibodies: E-cadherin (dilution 1:500; Abcam), N-cadherin (dilution 1:500; Abcam), Vimentin (dilution 1:250; Abcam), Slug (dilution 1:100; Cell Signaling), Ki67 (dilution 1:500; Abcam), and MMP9 (dilution 1:500; Abcam). This was followed by an incubation with a secondary antibody (dilution 1:500; Santa Cruz) for 2 h at 37 °C. The assessment of immunohistochemistry was performed using criteria established in previous studies [51, 52]. Immunostaining intensity was categorized into four distinct levels: 0 (negative), 1 (weak), 2 (moderate), and 3 (strong). The percentage of stained cells was categorized as follows: 1 (0–10%), 2 (11–50%), 3 (51–80%), and 4 (81–100%). The final immunohistochemistry score (IHS) was derived from the combined sum of the intensity and percentage scores. Histopathological evaluation (H&E staining, immunohistochemical scoring) was performed independently by two board-certified pathologists blinded to the treatment groups and experimental conditions.

### H&E Staining

Tumour specimens were fixed in a 4% paraformaldehyde solution for a duration of 24 h. Following fixation, the samples were subjected to a dehydration process and subsequently embedded in paraffin. Thin sections, each measuring 4 μm in thickness, were then prepared from the embedded specimens. The H&E staining procedure [39]was executed employing a commercially available kit procured from Beyotime (Shanghai, China; Catalogue No. C0105S). Post-staining, the samples were meticulously evaluated using an OLYMPUS BX53 microscope (Tokyo, Japan).

### Statistics

All in vitro experiments were performed in triplicate. Investigators performing data collection, outcome assessment, and initial analysis were blinded to group assignments. Data processing and figure plotting were performed using R (v4.3.1) software (http://www.R-project) and SPSS statistical software version 19.0 (SPSS Inc., Chicago, IL, USA), and *P* < 0.05 denoted the statistical significance.

## Results

### Identification and construction of DRLs

**Fig. 1:**
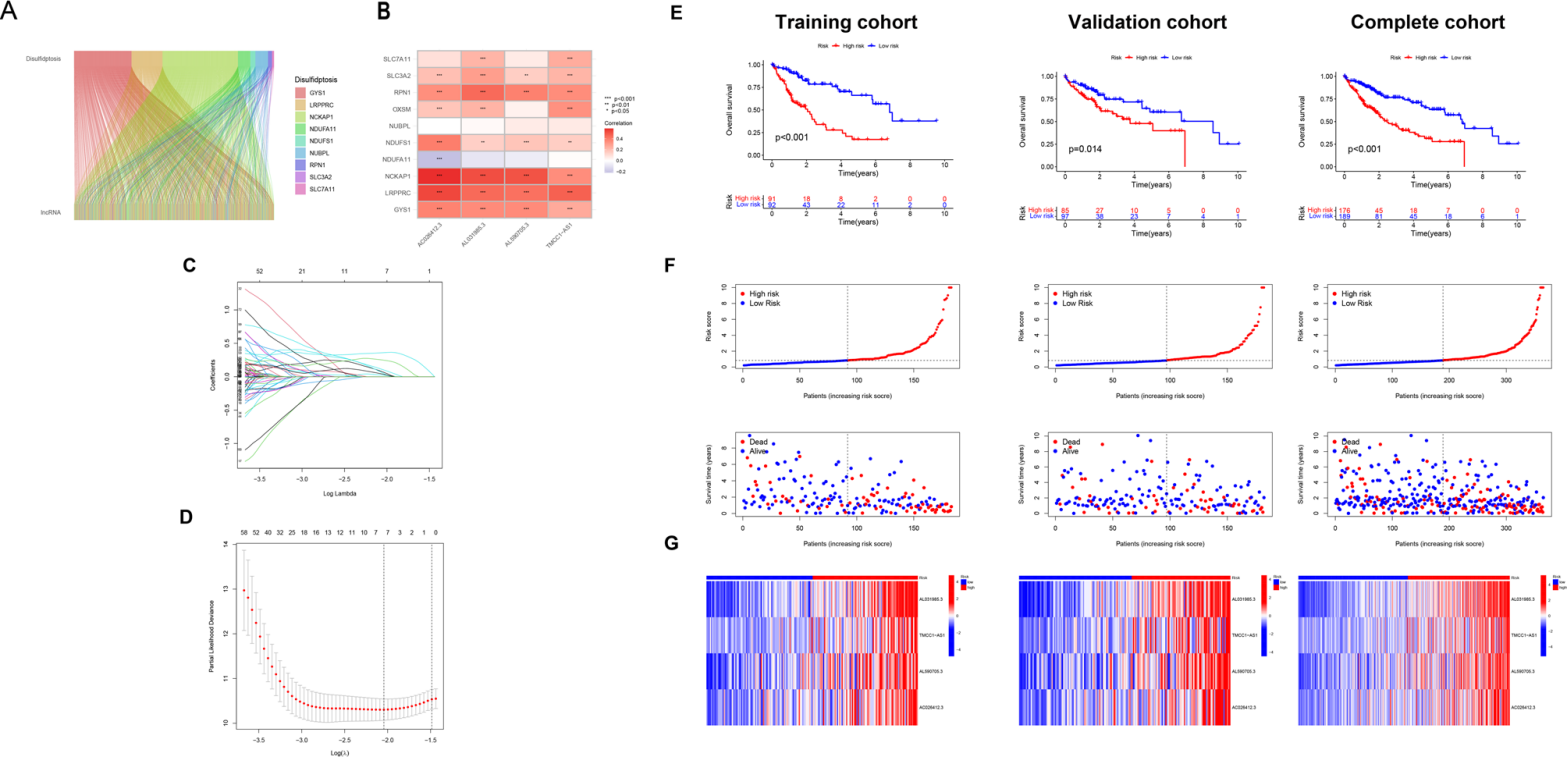
Construction of hepatocellular carcinoma (HCC) prognostic disulfidptosis-related lncRNAs (DRLs) risk model. **A** Sankey diagram illustrating the co-expression network between 9 disulfidptosis-related genes and 807 significantly correlated DRLs (*p* < 0.001, correlation coefficient > 0.4) identified in HCC. **B** Heatmap depicting the co-expression patterns (correlation coefficients) between 10 disulfidptosis-related genes and the 4 DRLs identified as independent prognostic factors. The colour scale represents the magnitude and direction of correlation (red: positive correlation, blue: negative correlation). **C** Prognostic prediction model constructed using least absolute shrinkage and selection operator (LASSO)-Cox regression analysis. LASSO coefficient profiles of HCC patients. **D** Ten-fold cross-validation for tuning parameter (lambda) selection in the LASSO Cox regression model. The partial likelihood deviance (mean ± standard error) is plotted against log(lambda). The vertical dotted lines denote the optimal lambda values chosen by the minimum deviance criterion (left line) and the one standard error rule (right line). **E** Kaplan-Meier (KM) curves comparing overall survival (OS) between high-risk and low-risk patient groups stratified by the 4-DRL signature risk score, within the training cohort, validation cohort, and the entire combined cohort. Log-rank p-values are indicated. **F** Distribution of risk scores (upper panel) and corresponding patient survival status (lower panel) for the training, validation, and entire cohorts. In the risk score distribution: patients are ordered by increasing risk score, red dots denote high-risk group classification, green dots denote low-risk group classification. In the survival status plot: red ticks indicate deceased patients, green ticks indicate living patients. **G** Heatmap visualising the expression levels of the 4 independent prognostic DRLs across patient samples in the training, validation, and entire cohorts. Red indicates relatively high expression, green indicates relatively low expression

We first identified 807 lncRNAs significantly co-expressed (*p* < 0.001, correlation coefficient > 0.4) with disulfidptosis-related genes in HCC (Fig. 1A). Patients were then randomly assigned to training and test cohorts, which exhibited comparable clinical characteristics (Supplementary Table 1). Using the training cohort, we constructed a DRLs prognostic model for HCC and subsequently validated it in the test cohort. Univariate Cox analysis identified 268 differentially expressed prognostic DRLs, as visualized in the forest plot (Supplementary Fig. 2). Subsequently, LASSO Cox regression analysis identified 7 DRLs with the highest prognostic value (*AC026401.3*,* AL031985.3*, *TMCC1-AS1*, *AL590705.3*, *MIR4435-2HG*,* AC026412.3*,* AC026356.1*; Fig. 1C, D). Multivariate Cox proportional hazards regression further identified four robust DRLs with independent prognostic significance. The risk score was calculated as follows: (Expression of *AL031985.3* × 0.5977) + (Expression of *TMCC1-AS1* × 0.5586) + (Expression of *AL590705.3* × 0.5639) + (Expression of *AC026412.3* × 0.7740). Figure 1B shows the correlation heatmap of the four prognostic DRLs and disulfidptosis-related mRNA.

**Fig. 2:**
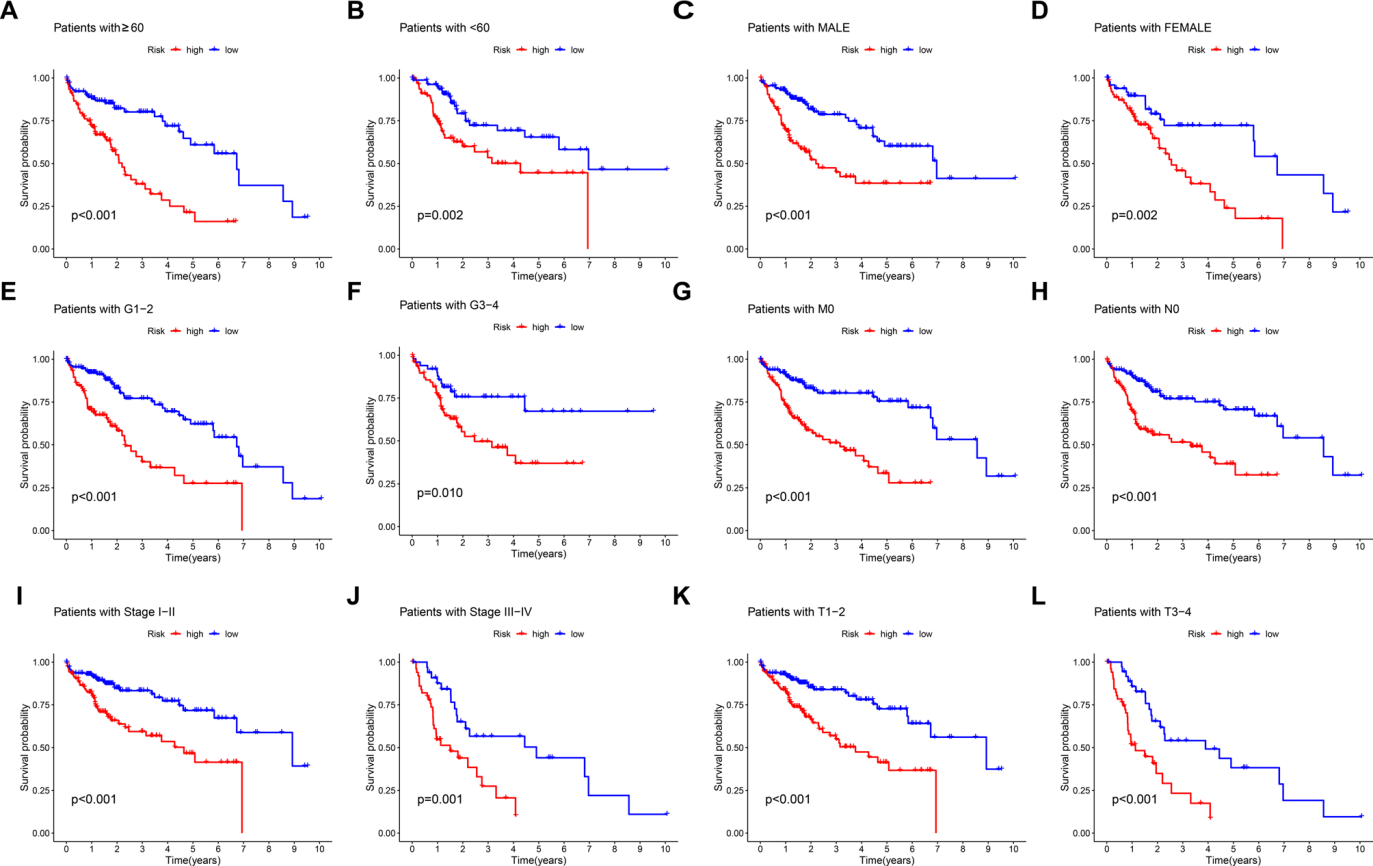
KM survival analysis according to the 4-DRL signature risk score across clinicopathological subgroups of HCC patients. KM curves illustrate the association between the 4-DRL signature risk group (high-risk vs. low-risk) and OS within distinct subgroups of HCC patients, stratified by: **A** age ≥ 60 years, **B** age < 60 years, **C** male, **D** female, **E** histological grade 1–2, **F** histological grade 3–4, **G** M0 stage (no distant metastasis), **H** N0 stage (no regional lymph node metastasis), **I** AJCC TNM stage I-II, **J** AJCC TNM stage III-IV, **K** T1-2 stage (primary tumour), and **L** T3-4 stage (primary tumour). Log-rank *p*-values are indicated for each comparison. Note: Comparisons for N1 and M1 stages were not performed due to insufficient patient numbers

Survival analysis demonstrated significantly lower OS in high-risk patients compared to low-risk patients across the training, validation, and entire cohorts (Fig. 1E). The distribution of risk scores and patient survival status are depicted in Fig. 1F. A heatmap illustrates the expression levels of the four DRLs across samples in the training, validation, and entire cohorts (Fig. 1G). Finally, to investigate the association between clinicopathological features and the OS risk score, HCC patients were stratified by gender, age, histopathological grade, TNM stage, T stage, N stage, and M stage. The findings demonstrated that, across most subgroups (except N1 and M1 stages, which lacked sufficient patient numbers for evaluation), the high-risk group exhibited significantly reduced OS compared to the low-risk group (Fig. 2A). These findings demonstrate that our 4-DRLs prognostic model effectively predicts the prognosis of HCC patients across diverse clinicopathological subgroups.

### Independent value of 4- DRLs signature in predicting OS

**Fig. 3:**
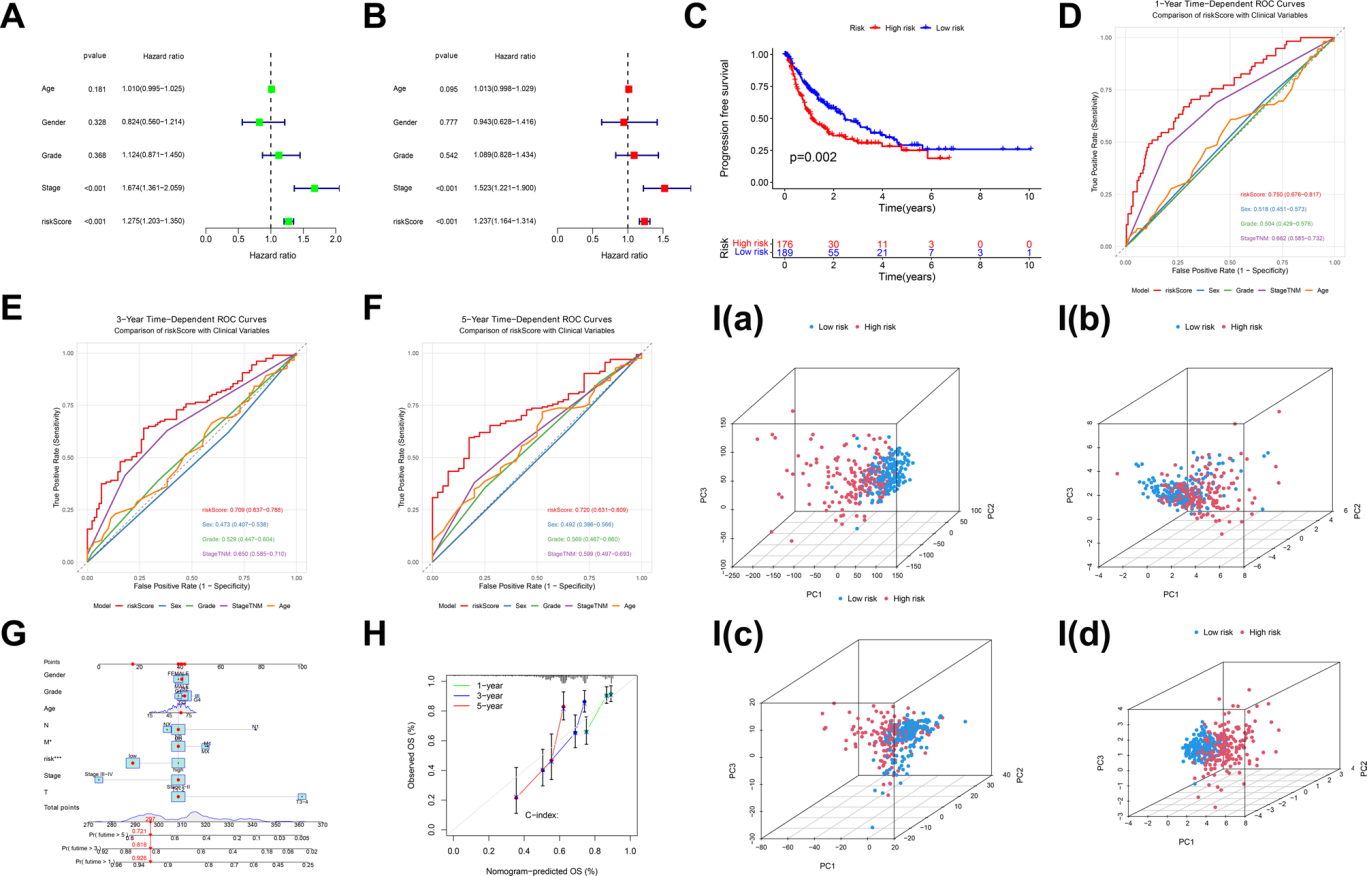
Independent prognostic analysis of HCC OS. **A** The univariate Cox proportional hazards analysis reveals statistically significant differences in survival outcomes, specifically highlighting the impact of stage and risk score on OS. **B** In the multivariate Cox analysis, both stage and risk score maintain their status as statistically significant prognostic indicators, underscoring their independent contributions to patient prognosis. **C** The KM survival curve illustrating progression-free survival (PFS) demonstrates a statistically significant difference between the two groups under investigation. **D** The 1-year Multiple Time Receiver Operating Characteristic (timeROC) curve analysis revealed that the predictive power of this prognostic model exceeds that of traditional clinical parameters, including sex, age, histopathological grade, and TNM stage. **E** The 3-year Multiple timeROC curve analysis revealed that the predictive power of this prognostic model exceeds that of traditional clinical parameters, including sex, age, histopathological grade, and TNM stage. **F** The 5-year Multiple timeROC curve analysis revealed that the predictive power of this prognostic model exceeds that of traditional clinical parameters, including sex, age, histopathological grade, and TNM stage. **G** Nomogram predicting prognosis based on the 4-DRLs signature score. To use the nomogram, choose a separate value on each variable axis and draw a straight line upward to determine the point value. The sum of all the value is located at the Total Points, and a straight line is drawn downward to obtain the probability of death within 1-year, 2-year, and 3-year for HCC patients. **H** The calibration curve indicates the goodness of fit of the nomogram. The abscissa of the graph is the predicted probability, which is the prediction of the likelihood of an event with the prediction model. The values 0 to 1 indicate a likelihood of an event of 0 to 100%. The ordinate is the actual probability and represents the actual incidence for the patient. Colored solid lines indicate the predictive performance of the nomogram. **I** To explore the differences between the low- and high-risk groups characterised by distinct expression profiles, we performed a principal component analysis (PCA). The results demonstrate that the model comprising the 4-DRLs used to construct the prognostic signatures (Id) effectively distinguishes between low-risk and high-risk HCC patients, outperforming models based on (Ia) all genes, (Ib) disulfideptosis-related genes, and (Ic) the DRLs prognostic model

Univariate and multivariate Cox regression analyses played a critical role in evaluating the independent predictive value of the prognostic model. Univariate Cox analysis identified TNM stage and the 4-DRLs risk score as significant prognostic factors (Fig. 3A). Multivariate Cox analysis confirmed both TNM stage and the risk score as independent predictors of OS (Fig. 3B). The KM analysis of PFS in the complete cohort demonstrated that the PFS significantly longer in the low-risk group compared to the high-risk group (*P* = 0.002, Fig. 3C). The multiple ROC analyses also confirmed that in the timeROC assessments at 1, 3, and 5 years, the predictive capability of this prognostic model surpasses that of other clinical indicators such as sex, age, histopathologic grade, and TNM stage (Fig. 3D–F). Eventually, we developed a prognostic nomogram (Fig. 3G) to estimate the 1-year, 3-year, and 5-year OS probabilities for individual patients. This nomogram incorporated five key predictors: the DRLs signature risk score alongside established clinical variables including patient age, gender, histopathological grade, and TNM stage. Each variable was assigned a specific point value on its respective axis. The total points, derived by summing the individual points for all five predictors, were then plotted on the ‘Total Points’ axis. A vertical line drawn downward from this total points value to the survival probability axes provided the estimated survival likelihood at the specified time intervals. Calibration curves (Fig. 3H) demonstrated strong concordance between nomogram-predicted and observed survival probabilities at 1, 3, and 5 years, indicating excellent calibration performance. We conducted PCA to explore the variations between the low- and high-risk groups with different expression spectra. The findings depicted that the prognostic DRLs used to construct the signature could reliably differentiate low- and high-risk HCC patients (Fig. 3Ia-d), further demonstrating the accuracy of the 4-DRLs model.

**Fig. 4:**
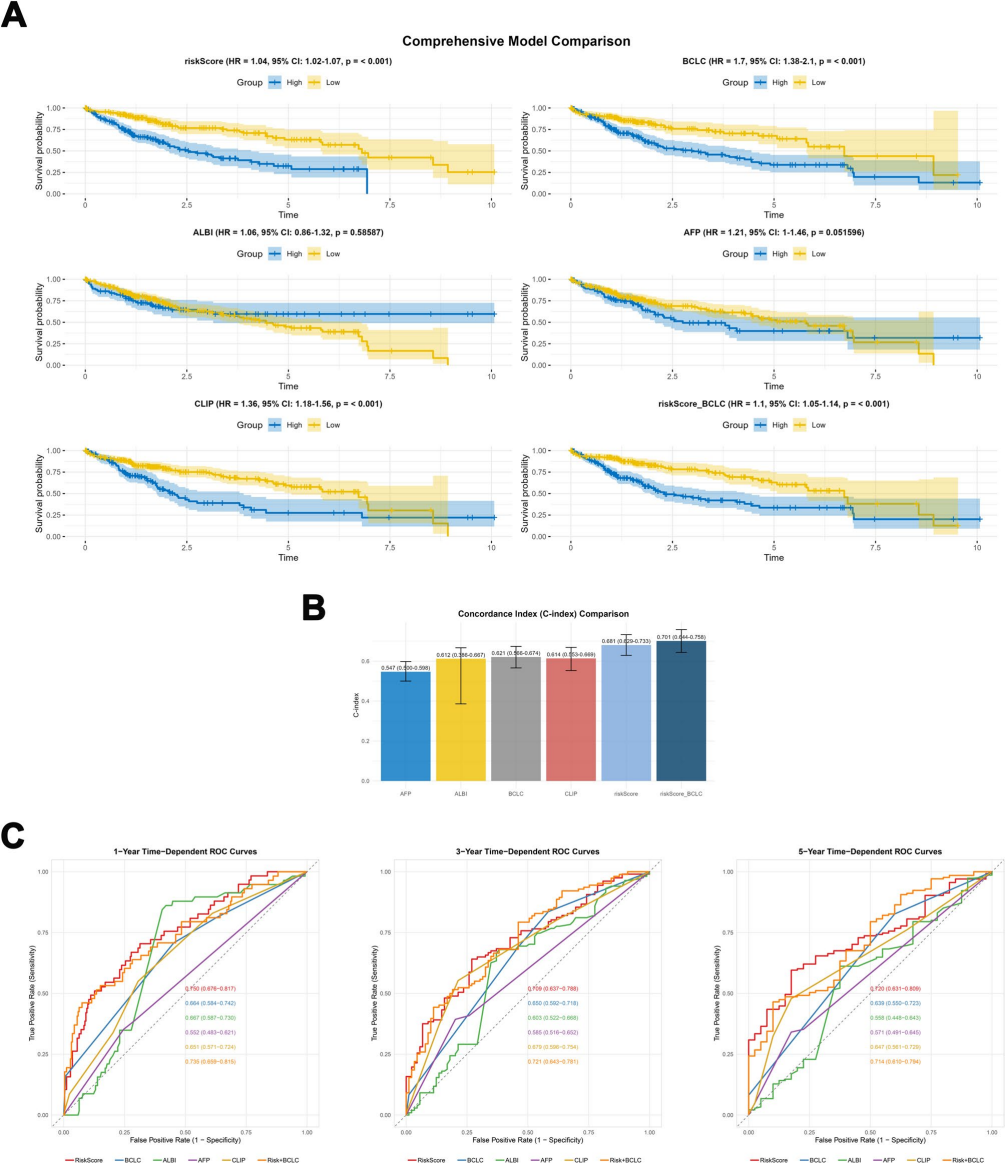
Comprehensive Model Comparison. **A** KM survival curve analysis comparing the RiskScore model with existing models, namely the Barcelona Clinic Liver Cancer (BCLC) Staging System, Albumin-Bilirubin (ALBI) Score, Cancer of the Liver Italian Program (CLIP) Score, alpha-fetoprotein (AFP) scoring system, and the combined RiskScore + BCLC model. **B** Comparative analysis of the concordance index (C-index) between the RiskScore model and the BCLC Staging System, ALBI Score, CLIP Score, AFP scoring system, and the combined RiskScore + BCLC model. **C** Comparative analysis of the time-dependent ROC curves at 1, 3, and 5 years between the RiskScore model and the BCLC Staging System, ALBI Score, CLIP Score, AFP scoring system, and the combined RiskScore + BCLC model

Furthermore, we conducted a comparison of the risk model with established traditional models such as the BCLC Staging System, ALBI Score, CLIP Score, and AFP scoring system, as well as with a combined model integrating our risk model with the BCLC system. KM analyses (Fig. 4A) revealed significant survival stratification by riskScore (HR: 1.04, 95% CI: 1.02–1.07, *p* < 0.001), with 5-year survival rates diverging markedly (low-risk:63.1% vs. high-risk:32.6%). The KM curve analysis for BCLC Staging System (HR: 1.7, 95% CI: 1.38–2.1, *p* < 0.001), CLIP Score (HR: 1.36, 95% CI: 1.18–1.56, *p* < 0.001), and combined RiskScore + BCLC model (HR: 1.1, 95% CI: 1.05–1.14, *p* < 0.001) also demonstrate commendable capacity for risk stratification. In contrast, ALBI (HR:1.06, 95% CI: 0.86–1.32, *p* = 0.586) and AFP (HR:1.21, 95% CI: 1-1.46, *p* = 0.052) failed statistical significance. Time-dependent ROC analysis demonstrated superior discriminative ability of riskScore over conventional systems across all intervals. At 1-year, riskScore achieved an AUC of 0.750 (95%CI: 0.676–0.817), exceeding BCLC (0.664; 0.584–0.742), ALBI (0.667; 0.587–0.730), AFP (0.552; 0.483–0.621), CLIP (0.651; 0.571–0.724), and the riskScore + BCLC hybrid (0.735; 0.659–0.815). This advantage persisted at 3 years (riskScore: 0.709 vs. BCLC: 0.650, ALBI: 0.603, AFP: 0.585, CLIP: 0.679, riskScore + BCLC: 0.721) and 5 years (0.720 vs. 0.639, 0.558, 0.571, 0.647, 0.714), with full curves presented in Fig. 4C. The riskScore + BCLC hybrid showed attenuated performance versus riskScore alone in metrics. Concordance (Fig. 4B) indices reinforced riskScore’s dominance (C-index:0.681, 95%CI: 0.629–0.733), surpassing BCLC (0.621; 0.566–0.674), ALBI (0.612; 0.386–0.667),,AFP(0.547; 0.500-0.598), and CLIP model (0.614; 0.553–0.669).

**Fig. 5:**
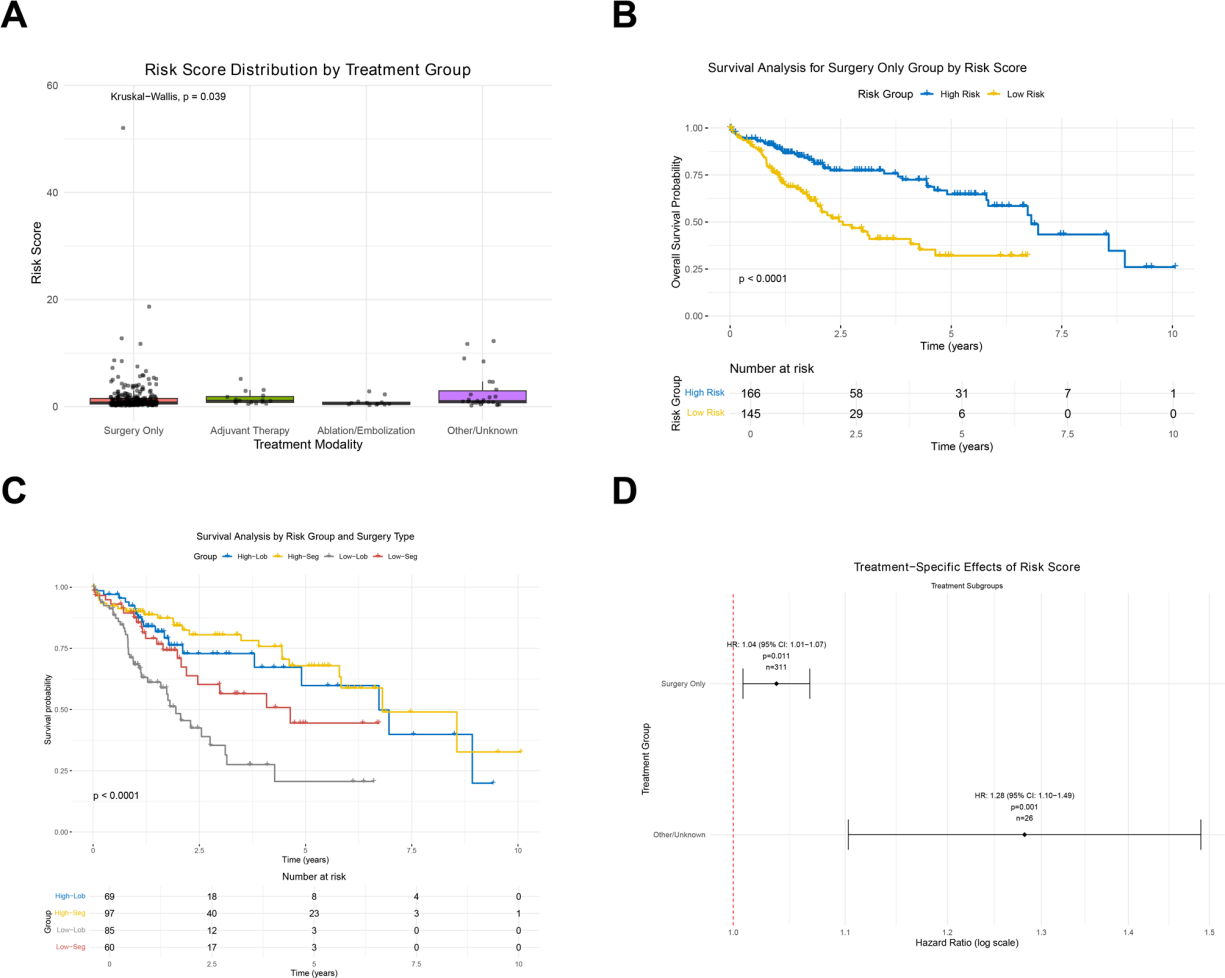
Heterogeneity analysis of the lncRNA-based risk signature across treatment modalities in the TCGA cohort. **A** Box plots depict the distribution of risk scores across treatment groups (Surgery Only, Adjuvant Therapy, Ablation/Embolization, and Other/Unknown). Significant heterogeneity was confirmed by the Kruskal-Wallis test (*p* = 0.0389), justifying further subgroup stratification. **B** Stratified KM survival curves for the Surgery Only cohort, demonstrating significant prognostic discrimination by the risk signature (log-rank *p* < 0.01). Curves are stratified by risk category (low vs. high). **C** Multilevel KM survival analysis of surgical subgroups within the Surgery Only cohort, stratified by procedure type (lobectomy vs. segmentectomy) and risk category (low vs. high). Low-risk patients (both segmentectomy and lobectomy) exhibit optimal survival, while high-risk lobectomy patients show the poorest outcomes. Number of patients at risk over time. Rapid attrition in high-risk subgroups reflects accelerated event rates. **D** The forest plot displays hazard ratios (HR) per unit increase in risk score for treatment groups with adequate sample size

In the forthcoming analysis, we will conduct a more detailed Treatment Heterogeneity Analysis. The Kruskal-Wallis test confirmed significant heterogeneity in risk score distribution among treatment groups (*p* = 0.0389; Fig. 5A). Clinical characteristics and median risk scores for each treatment group are summarized in Supplementary Table 2. Stratified KM analysis revealed robust prognostic discrimination by the risk signature within the Surgery Only cohort (log-rank *p* < 0.01; Fig. 5B). Further stratification of surgical subgroups by procedure type (lobectomy vs. segmentectomy) and risk category demonstrated markedly divergent survival outcomes: low-risk patients exhibited favourable prognosis irrespective of surgery type, whereas high-risk lobectomy patients suffered the poorest OS (5-year survival ≤ 5%; Fig. 5C). Risk-table analysis corroborated accelerated event rates in high-risk surgical subgroups, evidenced by rapid attrition (e.g., lobectomy high-risk: 85 to 3 patients at 5 years vs. low-risk: 69 to 8). Cox proportional hazards modelling with risk-score-by-treatment interaction terms identified a significant interaction for the Other/Unknown group (interaction coefficient = 0.251, *p* = 5.42 × 10⁻⁵; Supplementary Table 3). Hazard ratios for the lncRNA signature remained significant in larger subgroups: Surgery Only (HR = 1.037, 95% CI 1.008–1.067, *p* = 0.011) and Other/Unknown (HR = 1.282, 95% CI 1.103–1.489, *p* = 0.001; Fig. 5D). The elevated HR in Other/Unknown suggests enhanced prognostic utility within this cohort, though small sample sizes precluded reliable estimation in adjuvant and ablation subgroups. Collectively, these analyses confirm that the lncRNA signature retains prognostic value across heterogeneous treatments, with effect magnitude modulated by therapeutic modality—most notably exhibiting strengthened predictive capacity in incompletely documented treatment contexts.

### GO pathway enrichment analysis, GSEA, and PPI

**Fig. 6:**
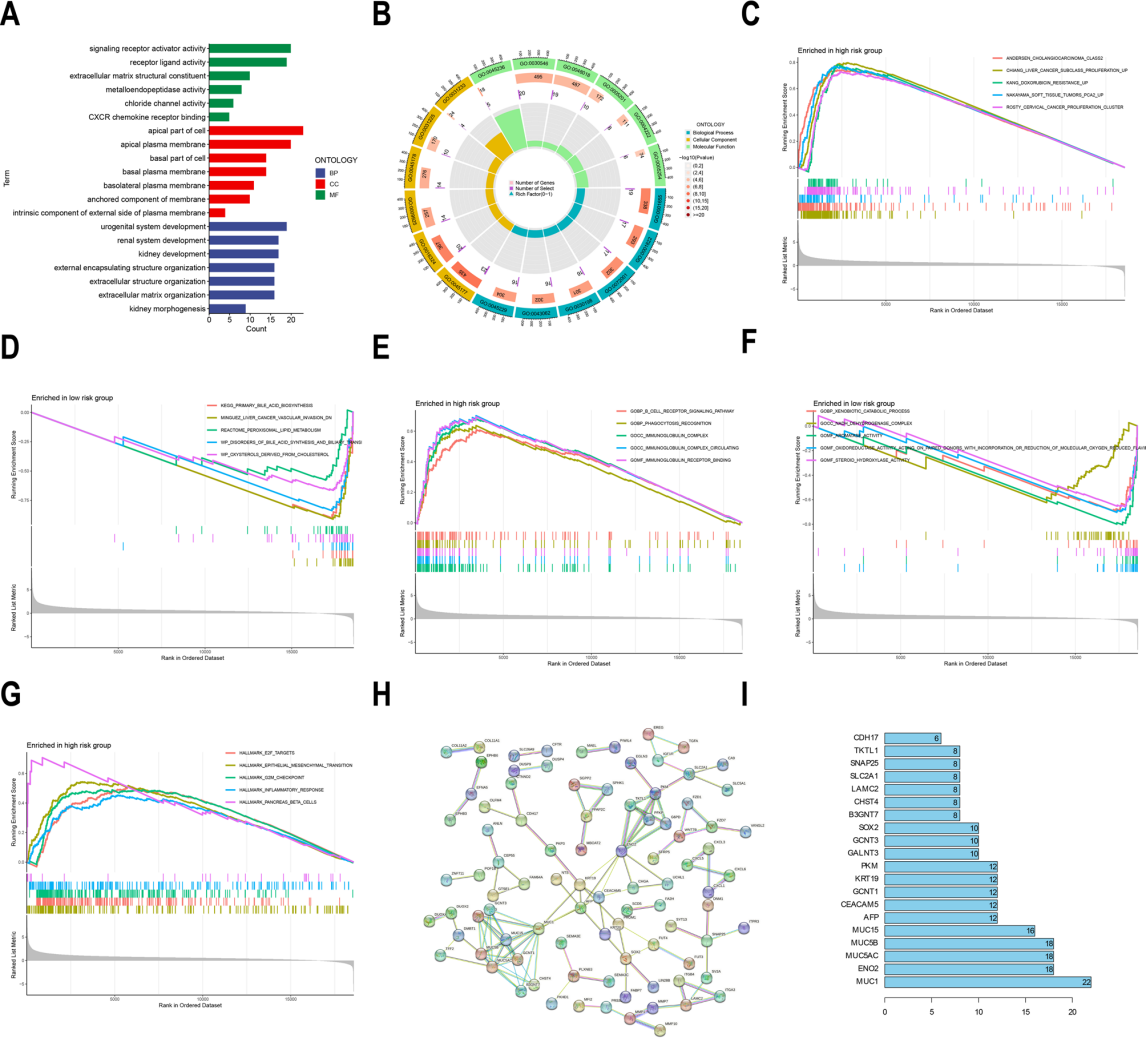
Gene ontology (GO), gene set enrichment analysis (GSEA), and protein-protein interaction (PPI) network. **A** The enrichment bar chart illustrates the GO analysis of differentially expressed genes between the high-risk and low-risk groups, as identified by the 4-DRLs risk model. The vertical axis represents the names of the GO terms, while the horizontal axis indicates the number of genes enriched in each biological pathway. Purple denotes biological processes (BP), red signifies cellular components (CC), and green represents molecular functions (MF). **B** The enrichment circle diagram presents the GO analysis of differentially expressed genes in the high and low risk groups identified by the 4-DRLs risk model. The plot utilises three distinct colour codes: teal, tawny, and bright green, which correspond to BP, CC, and MF, respectively. The first ring displays the top six GO terms for each category. The second ring illustrates the number of genes in the genomic background and the P-values for gene enrichment associated with the specified GO terms, where the colour intensity reflects the *P*-value for enrichment. The third ring denotes the number of differentially expressed genes enriched in the GO term. The fourth ring represents the enrichment factor for each GO term, indicating the proportion of genes. GSEA shows significant differences in enrichment in TCGA HCC cohort for the c2.all.v2022.1.Hs.symbols.gmt gene set between the 4-DRLs signature high-risk group (**C**) and low-risk group (**D**), and for the c5.all.v2022.1.Hs.symbols.gmt gene set between the high-risk group (**E**) and low-risk group (**F**). Significant enrichment in the h.all.v2022.1.Hs.symbols.cmt gene set was found in the high-risk group (**G**). The x-axis represents the ranked genes, while the y-axis indicates the enrichment scores. The curves in different colors correspond to distinct biological functions or pathways. **H** PPI network of differentially expressed genes (DEGs) between high and low-risk groups constructed using STRING. In the constructed PPI network graph, each node signifies a protein, while the lines delineate the associations between pairs of proteins. **I** Degree value information of nodes in the PPI network, highlighting the top 20 genes with the highest number of connections

Afterward, we conducted GO and GSEA enrichment analyses to further ascertain DEGs’ function analysis and biological pathway between the low- and high-risk groups of the 4- DRLs risk model. Using the Wilcox method, 310 DEGs were identified. These DEGs were primarily enriched in biological processes category pathways such as the development of the kidney, urogenital system, and renal system, as well as the organization of the extracellular structure and extracellular matrix, in the GO analysis (Fig. 6A and B). They were primarily enriched in the cellular component category in the apical plasma membrane, apical part of the cell, basal portion of the cell, and anchored component of membrane, basal plasma membrane, among others (Fig. 6A and B). They were significantly enriched in the molecule function category in CXCR chemokine receptor binding, receptor-ligand and signaling receptor activator activities, extracellular matrix structural constituent, and metalloendopeptidase activity, among other pathways and routes (Fig. 6 A and B). GO enrichment additionally revealed dysregulation of extracellular matrix organization and CXCR signaling in high-risk HCC. These pathways exacerbate actin cytoskeleton vulnerability—a core trigger of disulfidptosis—while promoting immune evasion. This mechanistic synergy underscores the 4- DRLs signature as a biomarker for tumors susceptible to disulfidptosis-inducing therapies (e.g., GLUT inhibitors) yet resistant to immunotherapy.

Further GSEA indicated that, in terms of the c2.all.v2022.1.Hs.symbols.gmt gene set, the high-risk patient group was remarkably involved in various biological pathways such as chiang liver cancer subclass proliferation up, andersen cholangiocarcinoma class2, nakayama soft tissue tumors pca2 up, kang doxorubicin resistance up, and rosty cervical cancer proliferation cluster (Fig. 6C). In addition, the low-risk patient group primarily participated in various pathways like kegg primary bile acid biosynthesis, wp disorders of bile acid synthesis and biliary transport, wp oxysterols derived from cholesterol, reactome peroxisomal lipid metabolism, and minguez liver cancer vascular invasion dn. (Fig. 6D). GSEA employing the c2.all.v2022.1.Hs.symbols.gmt curated gene set further revealed that disulfidoptosis in hepatocellular carcinoma is fundamentally orchestrated by hepatic metabolic reprogramming, notably suppression of bile acid biosynthesis (e.g., *CYP7A1/CYP27A1*). This metabolic perturbation synergises with proliferative dysregulation (*CDK1/TOP2A*) and stemness acquisition (*EPCAM/SOX9*), inducing oxidative stress and cytoskeletal destabilisation. Concurrent immune-evasive microenvironments, characterised by EMT signatures (*TWIST1/LOX*) and immunosuppressive cues, further potentiate disulfidoptosis susceptibility. These integrated pathways illuminate novel therapeutic targets for metabolic reprogramming in HCC. In terms of the c5.all.v2022.1.Hs.symbols.gmt gene set, both low- and high-risk patient groups participated in various BP, CC, and MF pathways. High-risk patient group mainly participated in gocc immunoglobulin complex circulating, gocc immunoglobulin complex, gomf immunoglobulin receptor binding, gobp phagocytosis recognition, and gobp b cell receptor signaling pathway, among others (Fig. 6E), while low-risk patient group mainly participated in gomf oxidoreductase activity acting on paired donors with incorporation or reduction of molecular oxygen reduced flavin or flavoprotein as one donor and incorporation of one atom of oxygen, gomf aromatase activity, gomf steroid hydroxylase activity, gobp xenobiotic catabolic process and gocc nadh dehydrogenase complex, among others (Fig. 6F). GSEA of the c5.all.v2022.1.Hs.symbols.gmt database further reveals profound dysregulation of oxidative stress and immune evasion pathways in HCC. Key findings demonstrate significant suppression of oxidoreductase activity (e.g., *CYP1A1*,* CYP3A4*), impairing detoxification and promoting disulfidoptosis through ROS-mediated disulfide stress. Concurrently, hyperactivation of B-cell-mediated immunity (e.g., immunoglobulin complexes; *IGHV* family) sustains an immunosuppressive tumour microenvironment, facilitating immune escape. Further exacerbating this phenotype, mitochondrial dysfunction (e.g., *NDUFB4*,* MT-ND1*) disrupts energy metabolism, accelerating oxidative damage. Therapeutically, targeting CYP450 enzymes to restore redox balance or inhibiting B-cell receptor signalling (e.g., SYK blockade) may counteract disulfidoptosis resistance and enhance immune surveillance in HCC. In terms of the h.all.v2022.1.Hs.symbols.gmt gene set, high-risk patient group mainly participated in hallmark epithelial mesenchymal transition, hallmark E2F targets, hallmark G2M checkpoint, hallmark inflammatory response and hallmark pancreas beta cells, among other epithelial-mesenchymal transition, checkpoint, inflammatory response, E2F targets pathways (Fig. 6G). No significant enrichment was encountered in the low-risk group. Through an in-depth GSEA analysis using the h.all.v2022.1.Hs.symbols.GSEA dataset, the results further revealed significant enrichment of EMT and glycolysis pathways. This suggests their roles in HCC metastasis and disulfidoptosis-related oxidative stress, thereby informing potential therapeutic targets. In the PPI network graph, nodes represent proteins and lines indicate associations between two proteins. By understanding the associations between protein molecules, the relationship between genes can be indirectly understood, thereby uncovering core regulatory genes. We developed a PPI network of risk DEGs utilizing STRING, which contained 91 nodes and 236 edges. There were 33 Hub genes with degree ≥ 5, and the top 20 genes with the most connections are shown in Fig. 6I. Among them, MUC1 is the most advanced gene (degree = 22) (Fig. 6H and I). The PPI network analysis identified key hub genes (e.g., MUC1, ENO2, SLC2A1) functionally linking disulfidptosis to HCC progression. MUC1’s centrality suggests its role in modulating disulfidptosis sensitivity alongside immune evasion, while glycolytic regulators (ENO2, PKM, SLC2A1) indicate metabolic adaptation to glucose stress—a disulfidptosis trigger. This implies targeting these hubs could therapeutically exploit disulfidptosis in HCC.

**Fig. 7:**
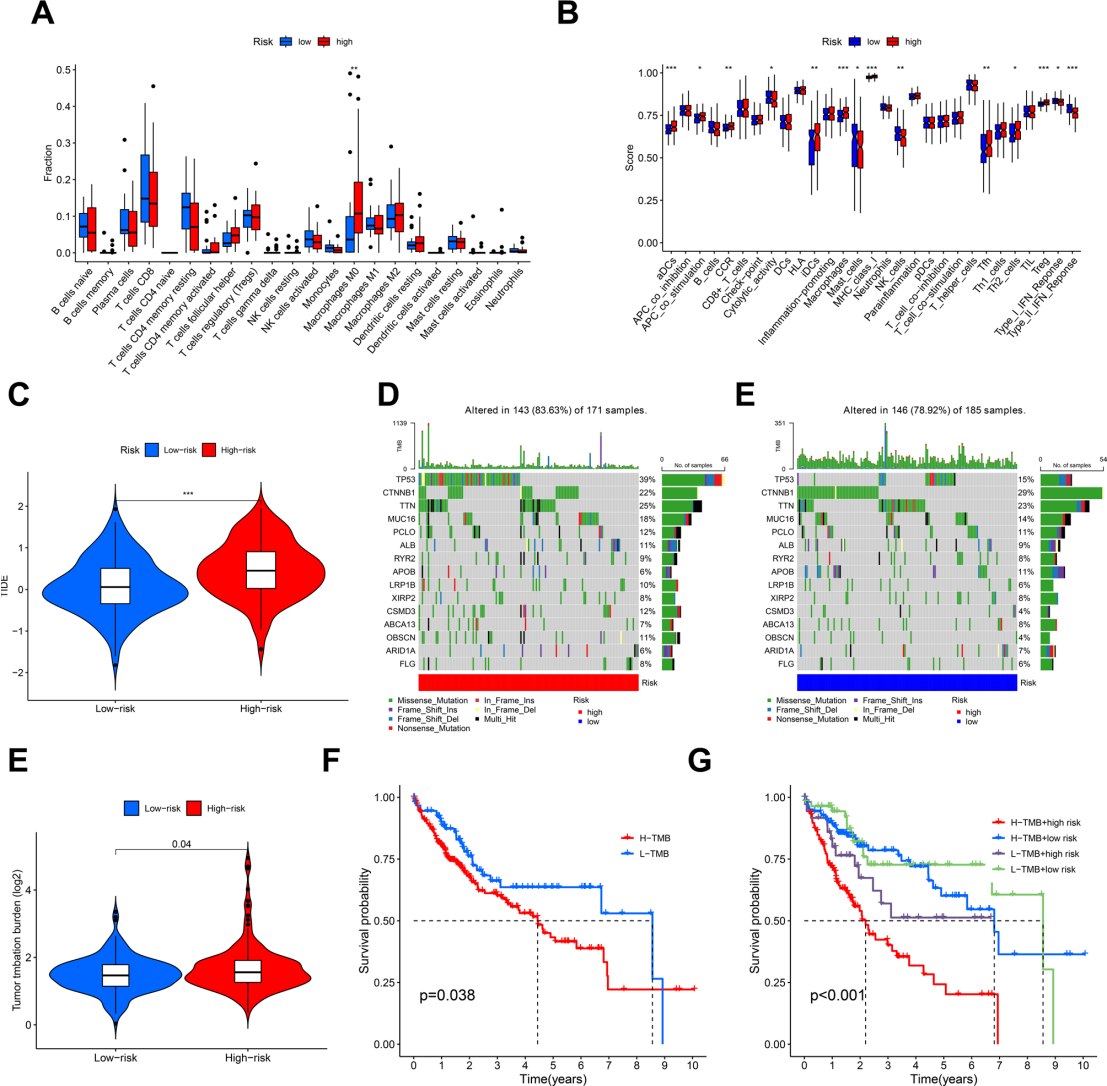
Immune landscape of HCC patients and relationship between tumor mutation burden (TMB) and risk score. **A** Percentage of 22 immune cells in high and low-risk groups calculated by CIBERSORT algorithm. The x-axis denotes the names of the immune cells, while the y-axis indicates the abundance of these immune cells. Blue bars correspond to samples from the low-risk group, and red bars correspond to those from the high-risk group. **B** Immune function scores of patients in high and low-risk groups. The x-axis represents immune-related functions, while the y-axis denotes the scores corresponding to these functions. Blue indicates samples from the low-risk group, whereas red denotes samples from the high-risk group. **C** Analyzing immune escape based on TIDE scores. The x-axis represents the risk groups of the samples, while the y-axis denotes the TIDE scores. Higher TIDE scores were observed in the high-risk group, suggesting a greater potential for immune evasion and consequently poorer outcomes from immunotherapy in this group. **D** Waterfall plot depicting mutations in the high-risk group. The x-axis represents the samples, while the y-axis denotes the gene names. Different colors indicate various mutation types. **E** Waterfall plot depicting mutations in the low-risk group. The x-axis represents the samples, while the y-axis denotes the gene names. Different colors indicate various mutation types. **F** The violin plot illustrating the differential analysis of TMB depicts variations in TMB between high and low-risk groups. The x-axis denotes the risk classification of the samples, while the y-axis represents the TMB values. The analysis reveals a statistically significant difference in TMB between the high and low-risk groups, with samples in the high-risk group exhibiting higher TMB levels. **G** KM survival analysis reveals a noteworthy distinction in prognostic outcomes between patient groups with different TMB. The analysis shows that patients in the low-TMB cohort have a significantly more favorable prognosis compared to those in the high-TMB cohort. **H** KM survival curves among the four distinct patient groups, categorized by their TMB and risk scores, indicate that patients with both low-risk scores and low TMB exhibited the most favorable prognosis. This group was closely followed by those with low-risk scores and high TMB. In contrast, patients with high-risk scores and low TMB had poorer prognostic outcomes. Notably, those with both high-risk scores and high TMB faced the most adverse prognosis. Significant differences are indicated by *P < 0.05, **P < 0.01, and ***P < 0.001

### Immune landscapes analysis between the 4-DRLs signature’s low and high-risk groups

CIBERSORT analysis revealed significantly reduced M0 macrophage infiltration in the low-risk versus high-risk cohort (Fig. 7A). The immune function scores of low-risk group patients, comprising Type I Interferon (IFN) Response, Cytolytic activity, and Type II IFN Response, NK cells, were significantly greater than the high-risk group, whereas the immune-related functions of APC co stimulation, CCR, aDCs, iDCs, MHC class I, Macrophages, Tfh, Th2 cells, and Treg showed the opposite pattern (Fig. 7B). Our findings reveal the variations in immune infiltration between the two groups. These variations in the immune microenvironment highlight the potential for immunotherapy. Consistent with immune dysfunction, TIDE scores confirmed increased immune escape potential in high-risk patients (*p* < 0.001, Fig. 7C).

### TMB of DRLs prognostic markers in HCC samples

Data on genetic mutations in patients with HCC were analyzed and the 15 genes with the highest mutation frequencies were selected for visualization. The top ten mutated genes in the low- and high-risk groups were *TP53*,* CTNNB1*,* TTN*,* MUC16*,* PCLO*,* ALB*,* RYR2*,* APOB*,* RP1B*, and *XIRP2* (Fig. 7D and E). The frequency of *TP53*,* MUC16*,* TTN*,* ALB*,* PCLO*,* LRP1B*, and *RYR2* mutations was significantly higher in patients in the high-risk group of the 4-DRLs model in contrast to those in the low-risk group. However, the mutation frequency of *CTNNB1* and *APOB* showed the opposite pattern (Fig. 7D, E). Additionally, we examined the TMB of each group and found that the high-risk group exhibited a greater TMB than the low-risk group (*P* = 0.04) (Fig. 7F). The K-M analysis further inferred that in contrast to the high TMB group, a better prognosis was reported by patients in the low-TMB group ((*P* = 0.038). This indicates that the low-risk group may gain benefit from immunotherapy (Fig. 7G). Combining TMB with patient risk scores, the survival rates of the four groups demonstrated that patients who had low-risk scores + low TMB had the best prognosis, followed by those who had low-risk scores + high TMB. Those with high-risk scores + low TMB and those with high-risk scores + high TMB had a worse and worst prognosis, correspondingly (Fig. 7H).

### Drug sensitivity analysis of hepatocellular carcinoma

**Fig. 8:**
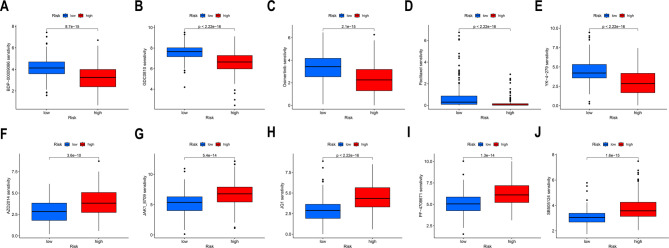
Drug sensitivity of HCC patients in high-risk and low-risk groups based on the 4- DRLs signature. The distribution of the half maximal inhibitory concentration (IC50) shows significant differences between patients in the low-risk group and high-risk group in **A** BDP-00009066, **B** GDC0810, **C** Osimertinib, **D** Paclitaxel, **E** YK-4-279, **F** AZD2014, **G** JAK1_8709, **H** JQ1, **I** PF-4708671, and **J** SB505124. Figures **A**–**E** illustrate that samples from the high-risk group exhibit lower scores and correspondingly higher sensitivity to the respective drugs. Conversely, figures **F**–**J** demonstrate that samples from the low-risk group have lower scores, indicating greater sensitivity to the respective drugs. The x-axis represents the risk stratification of the samples, while the y-axis denotes drug sensitivity

We used oncoPredict to select currently used chemotherapeutic and targeted drugs for treating HCC to ascertain the sensitivity of the two-risk groups of patients of the 4-DRLs signature to these drugs. The results demonstrated that high-risk group patients with elevated DRL scores were more sensitive to BDP-00009066, GDC0810, Osimertinib, Paclitaxel, and YK-4-279 (top five) (Fig. 8A–E), as indicated by lower IC50 values. Conversely, the IC50 values for drugs such as AZD2014, JAK1_8709, JQ1, PF-4,708,671, and SB505124 (top five) (Fig. 8F–J) were substantially lower in the low-risk group. In summary, these results indicate that these prognostic DRLs may be associated with drug sensitivity, providing clinical references.

### Analysis of risk prognostic lncRNA in 4-DRLs signature

**Fig. 9:**
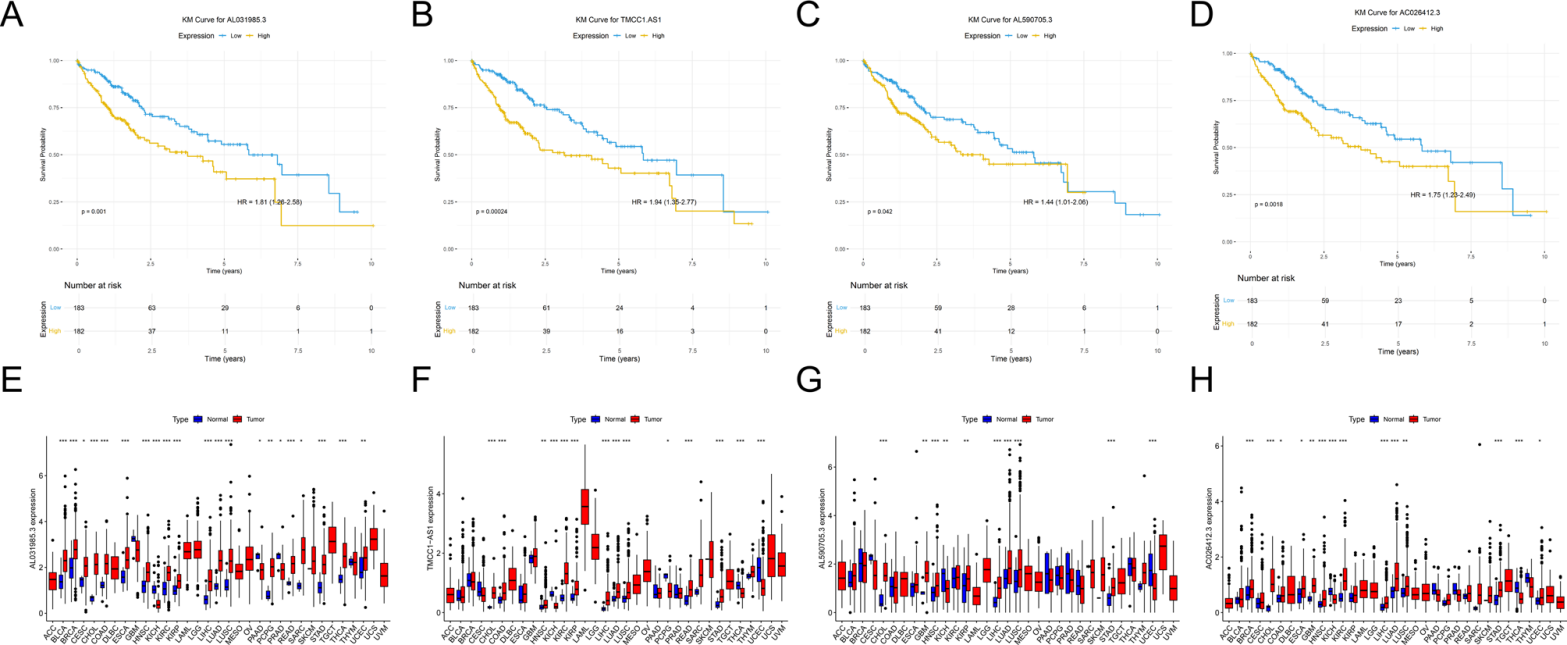
Analysis of prognostic risk LncRNAs in the 4-DRLs signature model. **A–D** The KM survival curve analysis was employed to evaluate OS differences between high-expression (red lines) and low-expression (blue lines) groups for the LncRNAs *AL031985.3*, *TMCC1-AS1*, *AL590705.3*, and *AC026412.3*. Elevated expression levels of these four prognostic DRLs within the model are statistically significantly associated with the prognosis of patients suffering from HCC, as evidenced by a p-value of less than 0.05. Patients exhibiting increased expression of *AL031985.3*, *TMCC1-AS1*, *AL590705.3*, and *AC026412.3* demonstrated diminished survival times. **E**–**H** A comprehensive pan-cancer analysis, encompassing 33 cancer types available from the TCGA database, was conducted to investigate the expression variations of four risk DRLs. This analysis identified differential expression patterns between tumour (red) and adjacent non-tumour tissues (blue) for *AL031985.3*,*TMCC1-AS1*, *AL590705.3*, and *AC026412.3*. Significant differences are indicated by *P < 0.05, **P < 0.01, and ***P< 0.001

We applied K-M to the 4-DRLs LncRNA *AL031985.3*, *TMCC1-AS1*, *AL590705.3*, and *AC026412.3*, and the results revealed that the high expression of the four prognostic DRLs in the model was linked to the prognosis of HCC patients (*p* < 0.05, Fig. 9A-D). Patients with high expression of *AL031985.3*, *TMCC1-AS1*, *AL590705.3*, and *AC026412.3* had shorter survival times. We delved deeper into the variation in the expression of these four risk LncRNAs in pan-cancer (33 types of cancer) that are available in the TCGA database. *AL031985.3* was remarkably lower in tumors such as kidney chromophobe, pancreatic cancer and prostate cancer in contrast to normal tissues, while its expression was upregulated than in normal tissues in cancers such as bladder cancer, cervical cancer, breast cancer, colon cancer, bile duct cancer and esophageal cancer (Fig. 9E). The expression of *TMCC1-AS1* was significantly lower in tumors such as kidney chromophobe, pheochromocytoma & paraganglioma and thyroid cancer than in normal tissues, while its expression was higher than in normal tissues in tumors such as bile duct cancer, colon cancer, head and neck cancer, kidney clear cell carcinoma, kidney papillary cell carcinoma and lung adenocarcinoma (Fig. 9F). The expression of *AL590705.3* was significantly lower than in normal tissues in tumors such as kidney chromophobe and endometrioid cancer, while its expression was remarkably elevated than in normal tissues in these tumors such as glioblastoma, lung adenocarcinoma, bile duct cancer, kidney papillary cell carcinoma, head and neck cancer, as well as lung squamous cell carcinoma (Fig. 9G). The expression of *AC026412.3* was markedly lower than in normal tissues in tumors such as kidney chromophobe and thyroid cancer. In comparison, its expression level was significantly higher than in normal tissues in these tumors like breast cancer, esophageal cancer, colon cancer, bile duct cancer, and glioblastoma (Fig. 9H).

**Fig. 10:**
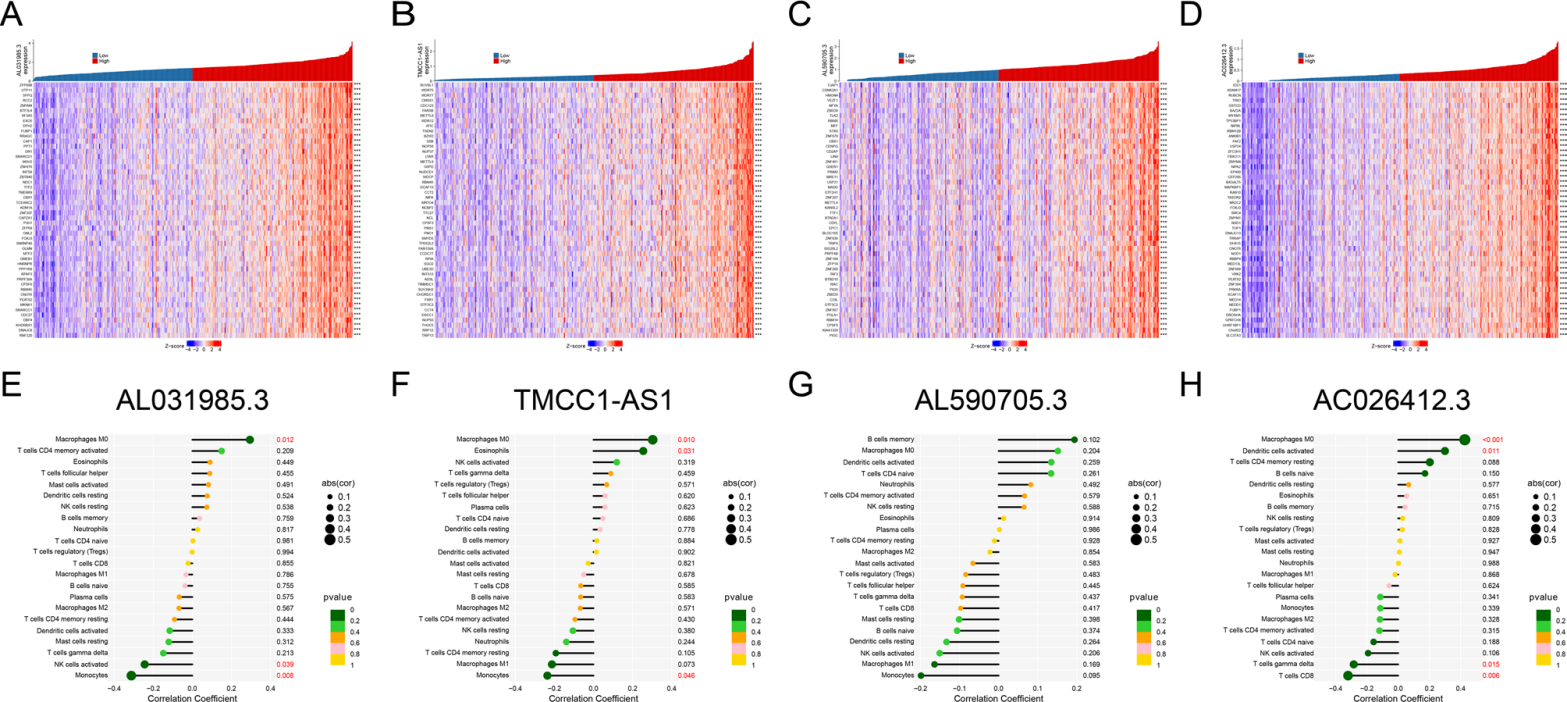
The expression level of four prognostic DRLs was associated with immune infiltration in the tumor microenvironment. The correlation heatmap shows the top fifty identified co-expression regulatory genes significantly related to *AL031985.3* (**A**), *TMCC1-AS1* (**B**), *AL590705.3* (**C**), and *AC026412.3* (**D**). The x-axis represents the sample groups, while the y-axis denotes the genes. Red indicates high expression levels, whereas green signifies low expression levels. **E** Correlation between the relative abundances of 22 immune cells and lncRNA *AL031985.3* expression level. A significant positive correlation was identified between macrophages M0 and the expression of lncRNA *AL031985.3* (*P* = 0.012). Conversely, a negative correlation was observed between the expression of *AL031985.3* and both monocytes (*P* = 0.008) and activated NK cells (*P* = 0.039). **F** Correlation between the relative abundances of 22 immune cells and lncRNA *TMCC1-AS1* expression level. Macrophages M0 (*P* = 0.010) and eosinophils (*P* = 0.031) were positively associated with *TMCC1-AS1* expression, whereas monocytes exhibited a negative association. **G** Correlation between the relative abundances of 22 immune cells and lncRNA *AL590705.3* expression level. There was no significant correlation between the expression of *AL590705.3* and the distinct immune cell types assessed. **H** Correlation between the relative abundances of 22 immune cells and lncRNA *AC026412.3* expression level. *AC026412.3* expression demonstrated a significant positive correlation with activated dendritic cells (*P* = 0.011) and macrophages M0 (*P*< 0.001), while showing a negative correlation with CD8+ T cells (*P* = 0.006) and gamma delta T cells (*P* = 0.015). Significant differences are indicated by***P < 0.001

Co-expression analysis of these four risk LncRNAs identified potentially regulated genes. The top fifty genes co-expressed with these four risk LncRNAs and the related heatmaps are shown in Fig. 10A-D. GO and GSEA analyses also revealed biological pathways related to these four risk LncRNAs (Supplementary Figs. 3–6). Spearman’s correlation coefficient determined the correlation between four prognostic DRLs and the infiltration levels of 22 immune cells. The macrophages M0 (*P* = 0.012) were significantly positively linked to *AL031985.3* expression. Monocytes(*P* = 0.008) and NK cells activated (*P* = 0.039) were negatively linked to lncRNA *AL031985.3* expression (Fig. 10E). As Fig. 10F demonstrates, Macrophages M0 (*P* = 0.010) and Eosinophils (*P* = 0.031) positively associated with *TMCC1-AS1*. However, Monocytes showed a negative association with *TMCC1-AS1*. *AC026412.3* expression was significantly positively correlated with Dendritic cells activated (*P* = 0.011) and Macrophages M0 (*P* < 0.001) and negatively linked to T cells CD8 (*P* = 0.006) and T cells gamma delta (*P* = 0.015) (Fig. 10H). As Fig. 10G demonstrates, no substantial correlation between *AL590705.3* expression and various types of immune cells. Supplementary Figs. 7–10 show the drug sensitivity of the four risk LncRNAs.

**Fig. 11:**
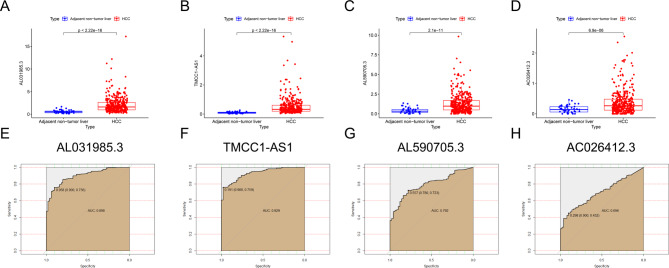
Differential expression and ROC of prognostic LncRNAs in TCGA HCC. **A–D** The expression levels of the four risk DRLs are significantly elevated in HCC specimens obtained from the TCGA cohort, when compared to the expression levels in adjacent non-cancerous tissue. **E–H** ROC analysis of HCC specimens obtained from the TCGA cohort demonstrated that *AL031985.3*,*TMCC1-AS1*1, *AL590705.3*, and *AC026412.3* exhibited favorable AUC values, indicating that these four risk DRLs possess significant diagnostic value for HCC

Box scatter plots affirm that the expression levels of 4 risk LncRNAs in TCGA HCC cohort specimens were remarkably greater than in para-cancerous normal tissues (Fig. 11A-D). Further ROC analysis (Fig. 11E-H) showed that the AUC of *AL031985.3* expression was 0.898 (95% CI:0.860–0.935, *P* < 0.001), with 90.0% sensitivity and 75.6% specificity. The AUC of *TMCC1-AS1* expression was 0.929 (95% CI: 0.900-0.958, *P* < 0.001), with 98.0% sensitivity and 75.9% specificity. The AUC of *AL590705.3* expression was 0.792 (95% CI:0.739–0.845, *P* < 0.001), with 78.0% sensitivity and 72.3% specificity. The AUC of *AC026412.3* expression was 0.696 (95% CI:0.634–0.758, *P* < 0.001), with 45.2% specificity and 90.0% sensitivity.

**Fig. 12:**
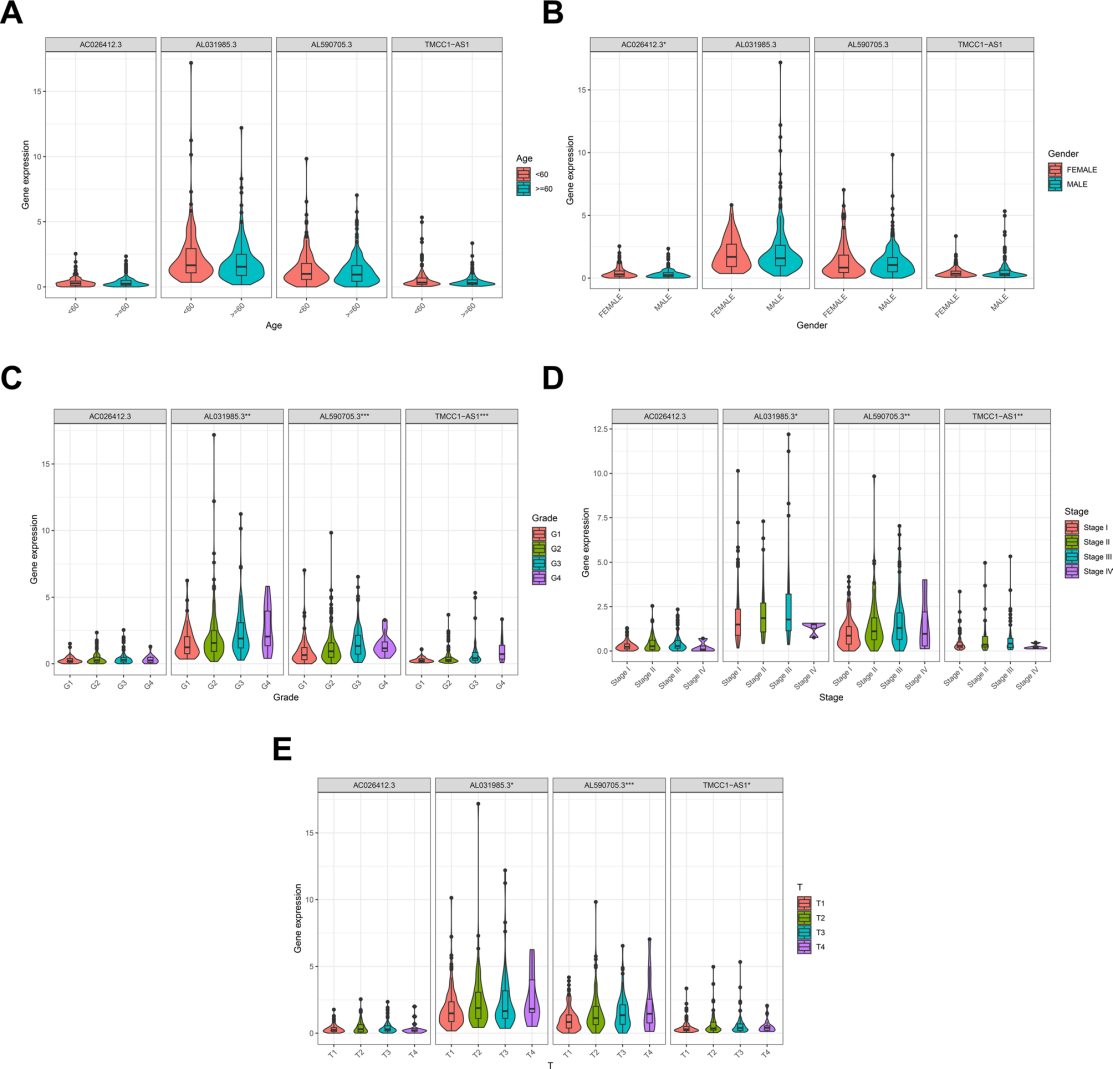
Association between expression levels of four prognostic DRLs and clinicopathological characteristics in HCC patients from the TCGA database. **A** Comparison of DRL expression between age cohorts (< 60 years vs. >= 60 years). **B** Comparison of DRL expression between male and female patients. **C** Comparison of DRL expression across pathological stages (G1, G2, G3, G4). **D** Comparison of DRL expression across TNM stages (I, II, III, IV). (E) Comparison of DRL expression across T-stage categories (T1, T2, T3, T4). Statistical significance was determined using the Wilcoxon rank sum test for two-group comparisons (**A**, **B**) and the Kruskal-Wallis test with Dunn’s post-hoc analysis for multi-stage comparisons (**C–E**). Asterisks denote significance thresholds: *P < 0.05, **P < 0.01, ***P < 0.001. Note that lncRNA *AC026412.3* exhibited significantly higher expression in females (**B**) but showed no significant association with pathological, TNM, or T stages (**C–E**). The remaining three DRLs demonstrated significant associations with pathological, TNM, and T stages, but not with age or gender

The Wilcoxon rank sum test method was also utilized to show the link between the expression level of 4 prognostic DRLs and the HCC clinicopathological characteristics. The findings imply that prognostic DRLs were substantially related to pathologic, TNM, and T stages, except for lncRNA *AC026412.3* (Fig. 12C-E). In addition, except for *AC026412.3*, which has higher expression in women, four diagnostic DRLs were not related to age and gender (Fig. 12A and B). Furthermore, we analysed expression data and clinical information of HCC from TCGA to assess the expression differences of the identified four DRLs across various etiologies, exploring their potential as etiology-specific biomarkers or therapeutic targets. Our findings indicate that the four DRLs are significantly upregulated in tumour tissues compared to adjacent normal tissues across all aetiologies examined—namely, Alcohol consumption (EtOH), Hepatitis B, Hepatitis C, and Non-Alcoholic Fatty Liver Disease (NAFLD) (Supplementary Figs. 11A-D). A comparison between groups with different etiologies demonstrated no significant expression differences among the DRLs across different etiologies (Supplementary Figs. 11E and G-J), except for *AL590705.3* (Supplementary Figs. 10F), which showed differential expression specifically between EtOH and Hepatitis C cases. These observations suggest that, while HCC may arise from different causative factors, the four DRLs associated with HCC prognosis might play a more fundamental or universal role in the disease’s progression rather than being directly linked to specific etiological factors. The consistent expression of these DRLs across different etiologies hints at their involvement in common molecular pathways or mechanisms, potentially affecting general cancer cell growth and proliferation rather than etiology-specific processes.

### Knockdown of AC026412.3 Inhibited the proliferation, invasion and migration of HCC cells

**Fig. 13:**
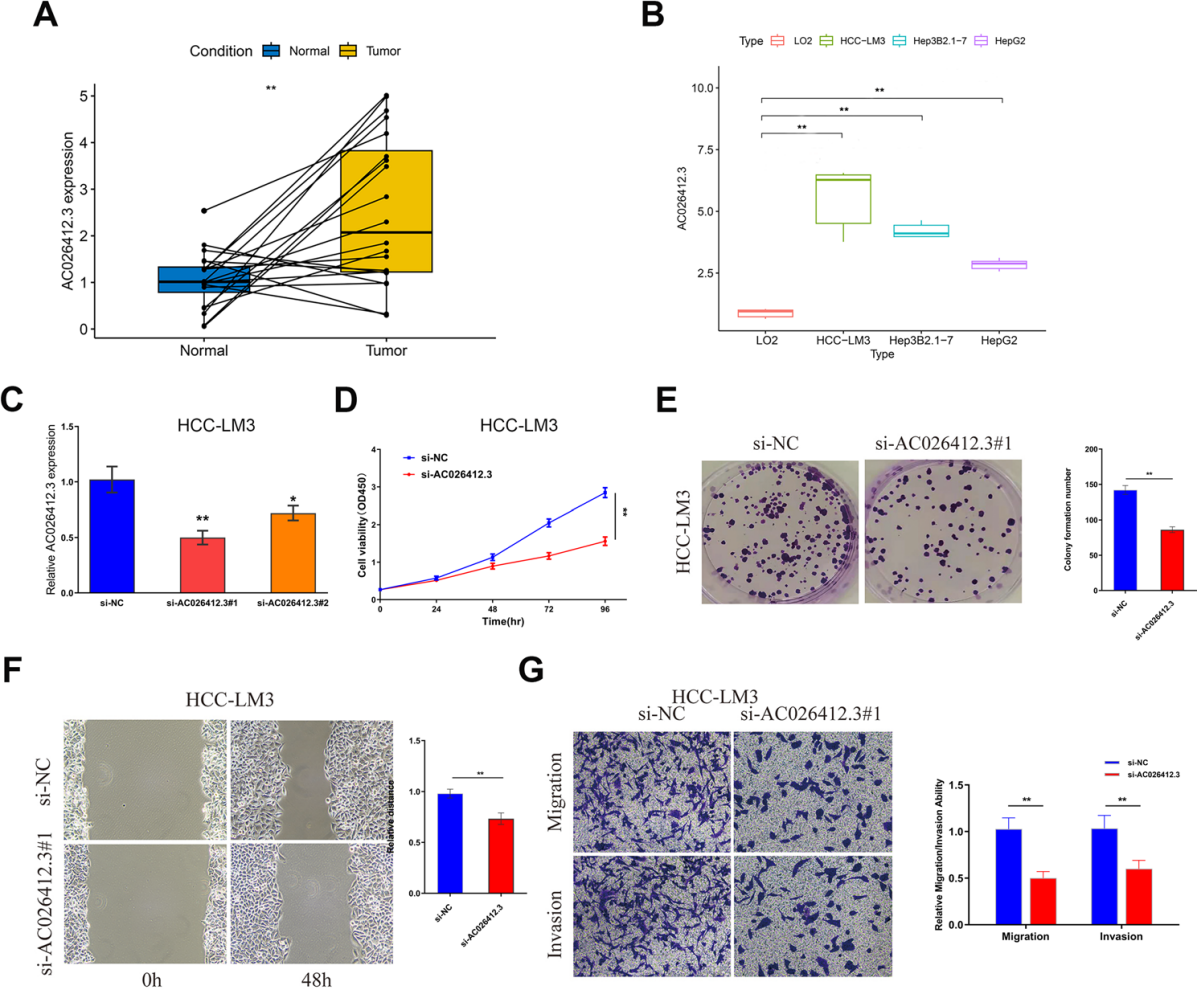
Functional experiments aginst *AC026412.3* in HCC cells.**A** Expression levels of *AC026412.3* in paired clinical samples of HCC and paracancerous tissues. Upon paired analysis, the expression of *AC026412.3* is significantly elevated in HCC tissues as compared to the corresponding paracancerous normal tissues. **B** Expression levels of *AC026412.3* in HCC cell lines HepG2, Hep3B2.1-7, HCC-LM3, and normal liver tissue LO2. *AC026412.3* was expressed at higher levels in all tested HCC cell lines relative to the LO2 cells. **C** Relative extression of *AC026412.3* after transfection with siRNAs. After RNA interference, the expression levels of the *AC026412.3* were significantly reduced. **D** The Cell Counting Kit-8 (CCK-8) assay revealed a marked reduction in proliferation following siRNA-mediated knockdown of *AC026412.3*. **E** The colony formation assay demonstrated a reduction in proliferative capacity following the disruption of *AC026412.3*. **F** The knockdown of *AC026412.3* led to a statistically significant decrease in the wound healing rate of HCC cells. **G** Transwell assays demonstrated a notable decline in cell invasion and migration capabilities post-knockdown. Significant differences are indicated by *P < 0.05 and **P < 0.01

Validation with clinical tumor samples showed that, in paired analysis, *AC026412.3* was significantly greater in HCC than in para-carcinoma normal tissues (Fig. 13A). To explore the expression mode of *AC026412.3* at the cellular level, we performed qRT-PCR in three HCC cell lines as well as one normal hepatic cell line LO2 (Fig. 13B). The findings revealed that in contrast to LO2, the expression of *AC026412.3* increased in all three HCC cell lines.

We performed functional experiments against *AC026412.3* in HCC cells. Firstly, the siRNAs were desigened and measured the knockdown efficiency by qRT-PCR (Fig. 13C). CCK-8 assay was performed to detect proliferation capacity and found that the proliferation capacity of *AC026412.3* was significantly reduced after transfection with the siRNAs (Fig. 13D). In the Colony formation assay, the ability of proliferation was significantly reduced after *AC026412.3* knockdown (Fig. 13E). Knockdown of *AC026412.3* resulted in a statistically significant reduction in the Wound healing rate of HCC cells (Fig. 13F). Transwell assays indecated diminished cell invasion and migration after *AC026412.3* knockdown (Fig. 13G).

### In vivo suppression of HCC progression and growth through LncRNA AC026412.3 silencing

**Fig. 14:**
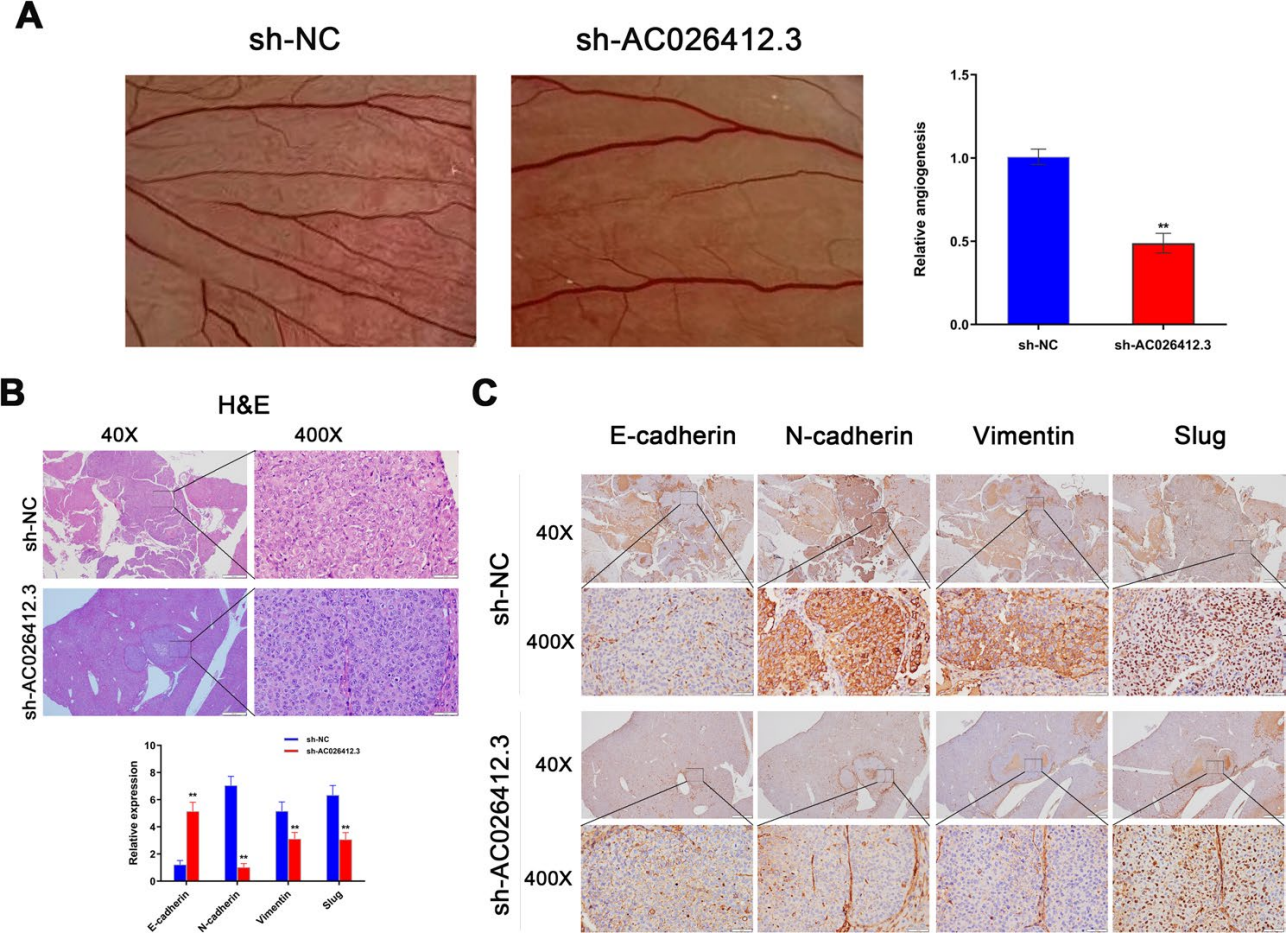
The effects of *AC026412.3* silencing on angiogenesis and epithelial-mesenchymal transition in HCC. **A** Analysis of angiogenesis using the chick chorioallantoic membrane (CAM) assay. A marked reduction in neovascularisation is observed in the group treated with sh-*AC026412.3* compared to the control group, indicating a significant impairment of angiogenic potential upon silencing of *AC026412.3*. **B** Histological examination through haematoxylin and eosin (H&E) staining within an orthotopic HCC model. At 40× magnification, tumour volume appears significantly reduced in the sh-*AC026412.3* group relative to the shNC control group. At a higher magnification of 400×, tumour cells in the sh-NC group display irregular, spindle-like morphology with extensive pleomorphism and stromal transformation, while cells in the sh-*AC026412.3* group exhibit more regular morphology with diminished pleomorphic characteristics. **C** Immunohistochemical analysis of epithelial and mesenchymal markers. E-cadherin, an epithelial marker, shows increased expression in the sh-*AC026412.3* group, whereas the mesenchymal markers N-cadherin, vimentin, and slug are predominantly expressed in the sh-NC group. This differential expression pattern suggests that *AC026412.3* silencing mitigates the epithelial-mesenchymal transition, contributing to a decrease in proliferative and metastatic capacity of HCC cells. Significant differences are indicated (**P < 0.01; two-tailed Student’s t-test)

To ascertain the in vivo function of *AC026412.3*, we initially employed the CAM assay to assess its impact on HCC angiogenesis. Our observations revealed a notable reduction in new blood vessel formation within the sh-*AC026412.3* group compared to the control group (Fig. 14A). Further validation was conducted using an orthotopic HCC model. Histological examination through HE staining showed that, at 40× magnification, tumour size was significantly larger in the shNC control group compared to the sh-*AC026412.3* group. At 400× magnification, tumour cells from the sh-NC group exhibited irregular, spindle-like morphology with marked pleomorphism and signs of stromal transformation. Conversely, cells from the sh-*AC026412.3* group displayed more regular morphology and reduced pleomorphism (Fig. 14B). Immunohistochemical analysis demonstrated elevated expression of the epithelial marker E-cadherin in the sh-*AC026412.3* group; in contrast, the mesenchymal markers N-cadherin, vimentin, and slug showed heightened expression in the sh-NC group (Fig. 14C). These findings collectively suggest that the silencing of *AC026412.3* impedes HCC cell proliferation and metastatic potential, thereby attenuating their capacity for epithelial-mesenchymal transition.

**Fig. 15:**
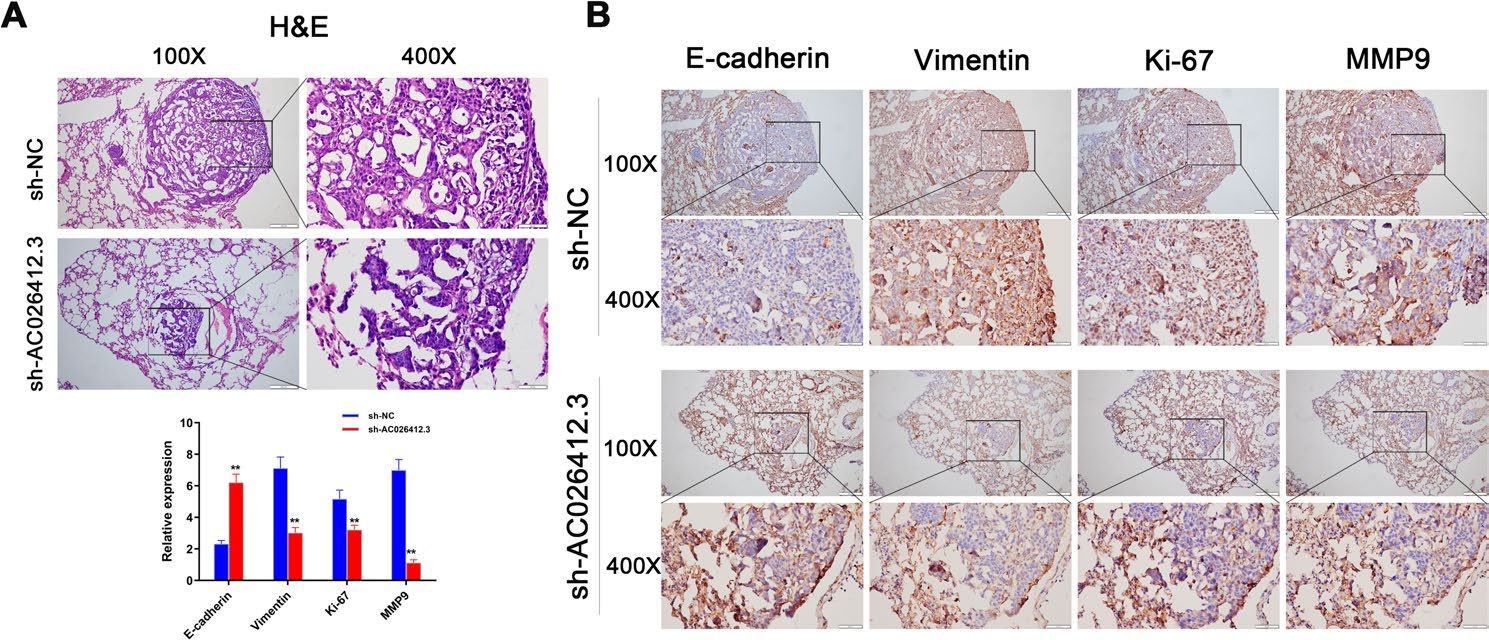
The Impact of LncRNA *AC026412.3* on lung metastasis and related molecular markers in a mouse model. **A** Comparative analysis of tumour cell infiltration in lung tissues of nude mice following tail vein injection of HCC cells transfected with either control vector (pL-NC-*AC026412.3*) or *AC026412.3*-knockdown vector (pL-sh-*AC026412.3*-LM3). H&E staining was employed to assess the extent of colonization. The results demonstrate a marked reduction in tumour cell infiltration in the pL-sh-*AC026412.3*-LM3 group compared to controls, highlighting the role of *AC026412.3* in promoting tumour growth, invasion, and metastasis. **B** Immunohistochemical analysis of key markers associated with tumour invasion and proliferation, including MMP9, E-cadherin, Vimentin, and Ki-67, in lung tissues from mice injected with pL-NC-LM3 or pL-sh-*AC026412.3*-LM3 cells. The IHC data reveal an upregulation of E-cadherin and a downregulation of Ki-67, Vimentin, and MMP9 in the pL-sh-*AC026412.3* group, corroborating the hypothesis that knockdown of *AC026412.3* attenuates cell proliferation and metastasis. Significant differences are indicated (**P < 0.01; two-tailed Student’s t-test)

Subsequently, the role of lncRNA *AC026412.3* in metastatic processes was examined using a mouse lung metastasis model, involving tail vein injection of either pL-sh-*AC026412.3*-LM3 or pL-NC-*AC026412.3* HCC cells. Tumour formation was observed within the lung tissues of nude mice across various groups. The extent of tumour cell colonization in the lungs was evaluated using H&E staining. A significant reduction in the infiltration of pL-sh-*AC026412.3*-LM3 tumour cells was observed relative to the control group (Fig. 15A), signifying that *AC026412.3* facilitates tumour growth, invasion, and metastasis. Considering that MMP9, E-cadherin, and Vimentin are linked to tumour invasion and metastasis — with Ki-67 serving as an indicator of cell proliferation — we verified their expressions in the pL-NC-LM3 and pL-sh-*AC026412.3*3-LM3 groups via IHC and qRT-PCR analyses. IHC results exhibited an increase in E-cadherin expression, alongside a decrease in Ki-67, Vimentin, and MMP9 expressions in lung tissues stably transfected with sh-*AC026412.3* (Fig. 13B). In conclusion, our data imply that *AC026412.3* augments the pulmonary metastasis of HCC cell lines by enhancing cell proliferation, tumour invasion, and metastatic capacity.

## Discussion

Recently, the concept of disulfidptosis was proposed, based on a rapid cell death caused by the emergency response to disulfide molecules induced by excessive accumulation of cysteine inside cells [29]. The mechanism of disulfide-triggered cell death is that in *SLC7A11*-greater cancer cells lacking glucose, an excessive buildup of disulfide molecules causes aberrant disulfide bonding between actin cytoskeleton proteins, interfering with their organization, eventually causing actin network collapse and cell death. However, research on DRLs in HCC is still scarce.

We identified DRLs in this present study and developed a prognostic signature comprising four DRLs (*AL031985.3*, *TMCC1-AS1*, *AL590705.3*, *AC026412.3*) using univariate Cox regression, LASSO regularization, and multivariate Cox regression. Univariate and multivariate analyses of COX indicated that the 4- DRLs risk score could act as an independent prognostic indicator for patients with HCC. The prognostic ROC, multi-ROC, C-index, KM survival curve, nomogram, and PCA results validated the reliability of this feature, suggesting that it has a promising predictive ability for HCC patients’ prognoses. Our findings demonstrate that the riskScore serves as a statistically robust prognostic tool, surpassing the established clinical systems such as BCLC, ALBI, CLIP, and AFP in predicting outcomes in patients with HCC. Despite methodological adaptations required for TCGA data—notably inferring BCLC stages from AJCC parameters and approximating CLIP’s portal thrombosis from vascular invasion records—the consistent superiority of riskScore across multiple validation frameworks underscores its translational potential. Unexpectedly, the combined riskScore + BCLC model showed lower AUC values compared to riskScore alone. This phenomenon likely stems from covariate redundancy between systems, as evidenced by moderate correlation (*r* = 0.48) and nonsignificant contribution of BCLC in multivariate Cox regression (HR = 1.12, *p* = 0.18). The limited discriminative capacity of BCLC staging within this cohort (C-index = 0.621) further suggests its prognostic information may be subsumed within the molecular signatures captured by riskScore. Notably, the elevated Akaike Information Criterion in the combined model (1624 vs. 1598) indicates suboptimal calibration when incorporating additional parameters without proportional predictive gain. Such divergence highlights fundamental differences in prognostic drivers: while BCLC reflects macroscopic pathological progression, riskScore encodes tumour microenvironment biology. Future integration efforts may warrant machine learning approaches to model nonlinear interactions, though our data affirm that molecular profiling alone provides superior prognostic stratification in contemporary HCC management. The model’s continuous risk stratification offers granularity unattainable by categorical systems like BCLC, particularly in intermediate-stage cohorts where treatment decisions remain contentious. Practical implementation is facilitated by the assay’s compatibility with Formalin-Fixed Paraffin-Embedded (FFPE) tissues via qRT-PCR, positioning riskScore as a feasible adjunct to routine histopathology. The cost-benefit advantage of lncRNA testing over whole-genome sequencing supports deployment in resource-limited settings. Future studies should validate its utility in prospective, different demographic and genetic backgrounds cohorts and explore synergies with emerging imaging biomarkers to refine personalised HCC management.

Further, we performed GO, GSEA, PPI, immune infiltration analysis, TMB, and drug sensitivity analysis on this feature. As per the GSEA, the DRLs risk model developed in this study is also linked to cell proliferation, vascular invasion, bile acid metabolism, epithelial-mesenchymal transition, and immune and inflammatory responses. Finally, the tumor microenvironment (TME) is closely linked to the risk model for HCC 4- DRLs. The findings indicated that the level of M0 macrophage infiltration in the high-risk group was significantly increased, implying that disulfidptosis may drive HCC progression via M0 macrophage recruitment. TIDE score can predict the response of patients to immunotherapy as it reflects the potential of tumor immune evasion. In our study, patients in the high-risk group showed higher TIDE scores, indicating a poorer response to immunotherapy. However, this conclusion requires validation by detailed in vivo and in vitro experiments in the future. Finally, TMB and drug sensitivity analyses provide a theoretical basis for immunotherapy, chemotherapy, or targeted drug treatment of HCC. Notably, our DRLs signature synergises with TMB to predict both HCC prognosis and immunotherapy response. High-risk patients exhibit elevated TIDE scores, indicating inherent resistance to ICIs likely mediated through lncRNA-driven immunosuppression, such as *AC026412.3*-associated M0 macrophage infiltration. This positions the 4- DRLs model as a dual biomarker for prognostic stratification and ICI response prediction. Clinically, high-risk patients may benefit from combinatorial therapeutic approaches targeting immunosuppressive cells alongside ICIs, whereas low-risk patients represent optimal candidates for ICI monotherapy. Our findings also position disulfidptosis as a critical interface between tumour metabolism and immune evasion. The accumulation of M0 macrophages in high-risk patients establishes an immunosuppressive milieu through T-cell inhibition and spatial exclusion mechanisms. Crucially, disulfidptosis susceptibility appears modulated by macrophage-derived cytokines, creating a reciprocal relationship that accelerates hepatocellular carcinoma progression. This crosstalk extends beyond macrophages as regulatory T cell infiltration and dendritic cell dysfunction collectively compromise adaptive immunity. Therapeutically, interventions targeting macrophage polarisation may simultaneously disrupt disulfidptosis-immune crosstalk and restore immunosurveillance.

The four lncRNAs in the model we constructed are all risk lncRNAs for HCC. We performed a detailed bioinformatics analysis on this and validated it with clinical HCC samples and HCC cell lines. Many lncRNAs in the signature have been proven to affect cancer prognosis. For example, *AL031985.3* is closely linked to the unfavorable prognosis of head and neck squamous cell carcinoma [53] and colorectal cancer [54]. More research indicates that *AL031985.3* is not only closely linked to the prognosis of HCC [55, 56]. Numerous studies [55, 57] have shown that *TMCC1-AS1* is associated with the OS rate and may serve as the prognostic biomarker for HCC individuals Some research has also shown that *TMCC1-AS1* is not only related to the OS rate of HCC but also to the relapse-free survival rate [58]. A nomogram constructed in combination with AFP and TNM, including the 4-lncRNA signature including *TMCC1-AS1*, can predict the early recurrence of HCC [59]. Chen et al. [60]. found that knocking down *TMCC1-AS1* can substantially inhibit the invasion, migration, and proliferation of SNU-182 and HepG2 cells while overexpressing *TMCC1-AS1* has the opposite effect. E-cadherin expression increases and proliferating cell nuclear antigen, Vimentin, N-cadherin, and Ki67 expression decreases in HepG2 cells when *TMCC1-AS1* expression is downregulated at the molecular level. Overexpressing *TMCC1-AS1* in SNU-182 cells has the opposite effect. These results indicate that the increase in the expression of *TMCC1-AS1* is not only linked to poor prognosis in patients with HCC, but it also promotes invasion, migration, proliferation, and the EMT process of HCC cells.

Currently, research concerning lncRNAs *AL590705.3* and *AC026412.3*, as well as their association with cancer, remains limited. *AC026412.3* appears as a crucial element in the lncRNA feature models related to HCC Necroptosis [28], cuproptosis [16, 61–63], Pyroptosis [64], cellular senescence [65], N1-Methyladenosine Methylation Regulators [66], 5-Methylcytosine Regulators [67]. The study by Xu et al. [68] elucidates that the upregulation of *AC026412.3* promotes proliferation of HCC cells and induces resistance to gefitinib, mirroring the findings of our current research. Our bioinformatics analysis suggests that *AC026412.3* may orchestrate immune evasion by recruiting M0 macrophages and suppressing cytotoxic T cells. This is mechanistically linked to disulfidptosis through *SLC7A11*-driven actin collapse, which releases DAMPs that activate NF-κB and chemokine pathways (e.g., *CXCL8/CCL20*). Consequently, M0 macrophages polarize toward an M2-like phenotype, secreting IL-10/TGF-β and expressing PD-L1 to inhibit T-cell function. This model aligns with our TIDE results, where high *AC026412.3* expression correlated with T-cell dysfunction and exclusion. We extensively explored the biological function of *AC026412.3* within HCC through in vivo, in vitro, and CAM assays. The CCK-8 assay, colony formation assay, wound healing assay, and Transwell assay collectively indicate that silencing *AC026412.3* inhibits the proliferation and migration of HCC cells. Furthermore, the CAM assay confirmed that the suppression of *AC026412.3* reduces its impact on endothelial cell proliferation and movement. Additionally, we established an orthotopic HCC model in nude mice and a HCC lung metastasis model; preliminary results from HE and IHC staining of the orthotopic HCC model suggest a correlation between *AC026412.3* and EMT. In the lung metastasis model exhibiting sh-*AC026412.3* transfection, there was observed upregulation of E-cadherin and downregulation of Ki-67, vimentin, and MMP9 within invasive lung tissues. These findings imply that *AC026412.3* enhances lung metastasis in HCC cell lines through promoting proliferation and the potential for tumour invasion and metastasis, thereby corroborating its role in facilitating EMT in HCC cells. The results propose that targeting *AC026412.3* might influence invasion, metastasis, and EMT of HCC cells by modulating intricate mechanisms, thus making it a promising target for HCC intervention strategies.

Although our prognostic feature model performs well in predicting HCC diagnosis, prognosis, and treatment response, and has been preliminarily validated by qRT-PCR and in vitro cell functional experiments, the research still has some limitations. Firstly, we acknowledge substantive limitations regarding the exclusive reliance on TCGA data. The cohort’s predominant derivation from academic centres in developed nations may introduce geographical and healthcare-access biases, potentially limiting the model’s generalisability to global hepatocellular carcinoma populations characterised by divergent aetiologies and socioeconomic determinants. Furthermore, inherent technical heterogeneity in TCGA sample processing—encompassing tissue collection protocols, RNA stabilisation methods, and sequencing batch effects—could systematically influence lncRNA quantification. While rigorous normalisation procedures and statistical thresholds (e.g., FDR correction,|cor| >0.4) were applied to mitigate analytical variability, the potential for protocol-driven artefacts in expression measurements remains non-negligible. Secondly, it is pertinent to acknowledge a significant limitation of this study concerning the external validation of our DRL prognostic signature. While robust internal validation was performed within the TCGA-LIHC cohort using a split-sample approach, efforts to validate the model in independent external cohorts encountered substantial practical constraints. Comprehensive searches of major public repositories, including multiple relevant Gene Expression Omnibus (GEO) datasets and International Cancer Genome Consortium (ICGC) datasets, revealed that none possessed expression data for all four signature lncRNAs (*AL031985.3*, *TMCC1-AS1*, *AL590705.3*, *AC026412.3*) coupled with sufficient survival information. This challenge likely stems from platform-specific differences in transcriptome coverage and annotation, particularly for less-characterised lncRNAs, and the variable focus of existing datasets. While this precludes immediate external validation using public data, we have endeavoured to strengthen the model’s credibility through rigorous internal validation, compelling functional validation of the key lncRNA *AC026412.3* in vitro and in vivo, and demonstration of the signature’s consistent prognostic performance across diverse HCC aetiologies within the TCGA cohort. We explicitly recognise the restricted generalisability imposed by the lack of independent cohort validation and consider this a priority for future research. Thirdly, Mechanistically, while our functional analyses and enrichment studies strongly suggest that *AC026412.3* is implicated in disulfidptosis through its role in cytoskeleton instability, as evidenced by its involvement in glutathione metabolism, ECM signaling, and redox imbalance, three other core lncRNAs, namely *TMCC1-AS1*, *AL590705.3*, and *AL031985.3*, also contribute to disulfidptosis susceptibility through the dysregulation of redox homeostasis (glutathione/NADPH), ECM remodeling, mitotic fidelity, and immune-metabolic crosstalk. We concur that direct verification through molecular pathway analyses—such as RNA immunoprecipitation (RIP), downstream effector detection, and rescue experiments—is essential. This will be addressed in our ongoing investigations. Mechanistically, we will conduct Chromatin Isolation by RNA Purification sequencing (ChIRP-seq)/RNA pulldowns to identify protein interactors of *AC026412.3* targeting cytoskeletal-EMT regulators, perform Clustered Regularly Interspaced Short Palindromic Repeats (CRISPR) screens for disulfidptosis-related genetic dependencies, and utilise longitudinal Patient-Derived Xenograft (PDX) models combining glucose restriction with *SLC7A11* inhibition to assess therapeutic synergy. Fourth, our model is based on lncRNA expression profiles primarily obtained from baseline tissue samples. Considering that lncRNA dynamics may be affected by disease progression or therapeutic pressures, and acknowledging the technical limitations in lncRNA detection, such as their low expression levels and susceptibility to degradation during sample processing, variability may be introduced. To address these challenges, we implemented rapid tissue freezing, verified RNA integrity, and conducted technical replication in qRT-PCR assays. Nonetheless, future multicentre studies employing standardised protocols and emerging technologies like single-molecule counting, alongside longitudinal studies tracking these DRLs throughout HCC progression (e.g., pre-/post-therapy, at recurrence), will be crucial to confirm their temporal stability and enhance their clinical applicability.

Fifth, although we conducted comprehensive treatment-stratified analyses, residual heterogeneity persists within the ‘Other/Unknown’ treatment category. This grouping likely encompasses diverse interventions (e.g., chemotherapy, radiotherapy, or supportive care) that could not be discretely classified due to limitations in the TCGA dataset annotation. Consequently, the prognostic effect observed in this subgroup should be interpreted as an aggregate estimate rather than a therapy-specific association. Despite FDR correction in initial screening and enrichment analyses, false-positive risks from multiple testing persist, particularly during univariate Cox screening where FDR was omitted for model-building. This was mitigated using penalised regression (LASSO Cox) with cross-validation for feature selection. Crucially, robust performance in internal validation cohorts supports the signature’s reliability. While our model adjusted for key clinicopathological variables (TNM stage, grade, etc.), unmeasured confounders (e.g., comorbidities, treatment history) inherent to TCGA’s retrospective design may influence prognosis. To address these limitations, we will conduct a definitive prospective multi-centre study incorporating diverse cohorts from varied healthcare settings, utilizing standardized protocols, contemporary RNA sequencing, and emerging technologies (including imaging biomarkers and single-molecule counting), alongside granular clinical and therapeutic annotations. This study is essential to rigorously validate the signature’s universality and clinical utility across different demographic and genetic backgrounds, refine its applicability for personalised HCC management, and dissect modality-specific treatment effects.

## Conclusion

We created a prognostic 4-DRLs model related to disulfidptosis, which provides guidance for screening high-risk groups, reveals their impact on the tumor microenvironment, clinical pathological features, and prognosis, and analyzes their potential wide-ranging regulatory mechanisms. We also identified the treatment tendencies of 4-DRLs model in chemotherapy or targeted therapy as well as immunotherapy. Finally, we explored the association between hub DRLs in the model and HCC, along with their influence on the malignant biological behavior of HCC cells, through comprehensive bioinformatics analysis and a series of meticulously conducted in vitro and in vivo experiments. These findings affirm the clinical importance of DRLs in HCC and convey new insights to guide personalized immunotherapy approaches for HCC patients.

## Supplementary Information

Supplementary Figure 1. Sample Size and Power Analysis for Clinical Studies.

Supplementary Figure 2. Univariate Cox regression forest plot identifies 268 Disulfidptosis-related lncRNAs (DRLs) significantly associated with the overall survival (OS) of hepatocellular carcinoma (HCC) patients.

Supplementary Figure 3. Gene Ontology (GO) and Gene Set Enrichment Analysis (GSEA) for AL031985.3. (A) Bar charts of the top 10 enriched Gene Ontology (GO) terms in the categories of Biological Processes (BP), Cellular Components (CC), and Molecular Functions (MF). The vertical axis represents the GO term names, while the horizontal axis denotes the number of genes enriched in each biological pathway. The color of the bars represents the significance of enrichment. (B) Bubble chart illustrating the top 10 enriched GO terms for BP, CC, and MF. The vertical axis represents the names of the GO terms, while the horizontal axis denotes the proportion of genes associated with each term. The size of the bubbles corresponds to the number of genes enriched in each GO term, and the color of the bubbles indicates the significance of the enrichment. (C) The enrichment circular chart represents the GO analysis of co-expressed genes associated with AL031985.3. The plot utilises three distinct colour codes: teal, tawny, and bright green, which correspond to BP, CC, and MF, respectively. The first ring displays the top six GO terms for each category. The second ring illustrates the number of genes in the genomic background and the P-values for gene enrichment associated with the specified GO terms, where the colour intensity reflects the P-value for enrichment. The third ring denotes the number of co-expressed genes enriched in the GO term. The fourth ring represents the enrichment factor for each GO term, indicating the proportion of genes. GSEA shows significant differences in enrichment in the TCGA HCC cohort for the c2.all.v2022.1.Hs.symbols.gmt gene set between the AL031985.3 high-expression group and low-expression group (D), and for the c5.all.v2022.1.Hs.symbols.gmt gene set between the AL031985.3 high-expression group and low-expression group (E). Significant enrichment in the h.all.v2022.1.Hs.symbols.cmt gene set was found in the AL031985.3 high-expression group (F). The x-axis represents the ranked genes, while the y-axis indicates the enrichment scores. The curves in different colors correspond to distinct biological functions or pathways.

Supplementary Figure 4. GO and GSEA for TMCC1-AS1. (A) Bar charts of the top 10 enriched GO terms in the categories of BP, CC, and MF. The vertical axis represents the GO term names, while the horizontal axis denotes the number of genes enriched in each biological pathway. The color of the bars represents the significance of enrichment. (B) Bubble chart illustrating the top 10 enriched GO terms for BP, CC, and MF. The vertical axis represents the names of the GO terms, while the horizontal axis denotes the proportion of genes associated with each term. The size of the bubbles corresponds to the number of genes enriched in each GO term, and the color of the bubbles indicates the significance of the enrichment. (C) The enrichment circular chart represents the GO analysis of co-expressed genes associated with TMCC1-AS1. The plot utilises three distinct colour codes: teal, tawny, and bright green, which correspond to BP, CC, and MF, respectively. The first ring displays the top six GO terms for each category. The second ring illustrates the number of genes in the genomic background and the P-values for gene enrichment associated with the specified GO terms, where the colour intensity reflects the P-value for enrichment. The third ring denotes the number of co-expressed genes enriched in the GO term. The fourth ring represents the enrichment factor for each GO term, indicating the proportion of genes. GSEA shows significant differences in enrichment in the TCGA HCC cohort for the c2.all.v2022.1.Hs.symbols.gmt gene set between the TMCC1-AS1 high-expression group and low-expression group (D), and for the c5.all.v2022.1.Hs.symbols.gmt gene set between the TMCC1-AS1 high-expression group and low-expression group (E). Significant enrichment in the h.all.v2022.1.Hs.symbols.cmt gene set was found in the TMCC1-AS1 high-expression group (F). The x-axis represents the ranked genes, while the y-axis indicates the enrichment scores. The curves in different colors correspond to distinct biological functions or pathways.

Supplementary Figure 5. GO and GSEA for AL590705.3. (A) Bar charts of the top 10 enriched GO terms in the categories of BP, CC, and MF. The vertical axis represents the GO term names, while the horizontal axis denotes the number of genes enriched in each biological pathway. The color of the bars represents the significance of enrichment. (B) Bubble chart illustrating the top 10 enriched GO terms for BP, CC, and MF. The vertical axis represents the names of the GO terms, while the horizontal axis denotes the proportion of genes associated with each term. The size of the bubbles corresponds to the number of genes enriched in each GO term, and the color of the bubbles indicates the significance of the enrichment. (C) The enrichment circular chart represents the GO analysis of co-expressed genes associated with AL590705.3. The plot utilises three distinct colour codes: teal, tawny, and bright green, which correspond to BP, CC, and MF, respectively. The first ring displays the top six GO terms for each category. The second ring illustrates the number of genes in the genomic background and the P-values for gene enrichment associated with the specified GO terms, where the colour intensity reflects the P-value for enrichment. The third ring denotes the number of co-expressed genes enriched in the GO term. The fourth ring represents the enrichment factor for each GO term, indicating the proportion of genes. GSEA shows significant differences in enrichment in the TCGA HCC cohort for the c2.all.v2022.1.Hs.symbols.gmt gene set between the AL590705.3 high-expression group and low-expression group (D), and for the c5.all.v2022.1.Hs.symbols.gmt gene set between the AL590705.3 high-expression group and low-expression group (E). Significant enrichment in the h.all.v2022.1.Hs.symbols.cmt gene set was found in the AL590705.3 high-expression group (F). The x-axis represents the ranked genes, while the y-axis indicates the enrichment scores. The curves in different colors correspond to distinct biological functions or pathways.

Supplementary Figure 6. GO and GSEA for AC026412.3. (A) Bar charts of the top 10 enriched GO terms in the categories of BP, CC, and MF. The vertical axis represents the GO term names, while the horizontal axis denotes the number of genes enriched in each biological pathway. The color of the bars represents the significance of enrichment. (B) Bubble chart illustrating the top 10 enriched GO terms for BP, CC, and MF. The vertical axis represents the names of the GO terms, while the horizontal axis denotes the proportion of genes associated with each term. The size of the bubbles corresponds to the number of genes enriched in each GO term, and the color of the bubbles indicates the significance of the enrichment. (C) The enrichment circular chart represents the GO analysis of co-expressed genes associated with AC026412.3. The plot utilises three distinct colour codes: teal, tawny, and bright green, which correspond to BP, CC, and MF, respectively. The first ring displays the top six GO terms for each category. The second ring illustrates the number of genes in the genomic background and the P-values for gene enrichment associated with the specified GO terms, where the colour intensity reflects the P-value for enrichment. The third ring denotes the number of co-expressed genes enriched in the GO term. The fourth ring represents the enrichment factor for each GO term, indicating the proportion of genes. GSEA shows significant differences in enrichment in the TCGA HCC cohort for the c2.all.v2022.1.Hs.symbols.gmt gene set between the AC026412.3 high-expression group and low-expression group (D), and for the c5.all.v2022.1.Hs.symbols.gmt gene set between the AC026412.3 high-expression group and low-expression group (E). Significant enrichment in the h.all.v2022.1.Hs.symbols.cmt gene set was found in the AC026412.3 high-expression group (F). The x-axis represents the ranked genes, while the y-axis indicates the enrichment scores. The curves in different colors correspond to distinct biological functions or pathways.

Supplementary Figure 7. Drug sensitivity in HCC patients with high and low expression of AL031985.3. The x-axis represents groups with high and low expression levels of AL031985.3, while the y-axis indicates drug sensitivity.

Supplementary Figure 8. Drug sensitivity in HCC patients with high and low expression of TMCC1-AS1. The x-axis represents groups with high and low expression levels of TMCC1-AS1, while the y-axis indicates drug sensitivity.

Supplementary Figure 9. Drug sensitivity in HCC patients with high and low expression of AL590705.3. The x-axis represents groups with high and low expression levels of AL590705.3, while the y-axis indicates drug sensitivity.

Supplementary Figure 10. Drug sensitivity in HCC patients with high and low expression of AC026412.3. The x-axis represents groups with high and low expression levels of AC026412.3, while the y-axis indicates drug sensitivity.

Supplementary Figure 11. Expression patterns of four DRLs in HCC of different aetiologies based on data from The Cancer Genome Atlas (TCGA). (A) Comparative analysis of four DRLs expression between HCC tissues with alcohol consumption (EtOH) as the aetiological factor and adjacent normal tissues. (B) Comparative analysis of four DRLs expression in HCC tissues with hepatitis B as the aetiological factor versus adjacent normal tissues. (C) Comparative analysis of four DRLs expression in HCC tissues with hepatitis C as the aetiological factor versus adjacent normal tissues. (D) Comparative analysis of four DRLs expression in HCC tissues with Non-Alcoholic Fatty Liver Disease (NAFLD) as the aetiological factor versus adjacent normal tissues. (E) Comparative expression analysis of four DRLs between ethanol-induced and hepatitis B-induced HCC. (F) Comparative expression analysis of four DRLs between ethanol-induced and hepatitis C-induced HCC. (G) Comparative expression analysis of four DRLs between ethanol-induced and NAFLD-induced HCC. (H) Comparative expression analysis of four DRLs between hepatitis B-induced and hepatitis C-induced HCC. (I) Comparative expression analysis of four DRLs between hepatitis B-induced and NAFLD-induced HCC. (J) Comparative expression analysis of four DRLs between hepatitis C-induced and NAFLD-induced HCC.

Supplementary Table 1. Features of hepatocellular carcinoma (HCC) patients in the complete cohort, training cohort, and validation cohort.

Supplementary Table 2. Clinical characteristics and median risk score distribution across treatment groups.

Supplementary Table 3. Cox proportional hazards model with interaction terms.

Supplementary Material 15.

## Data Availability

All data generated or analyzed are included in this article. Further inquiries can be directed to the corresponding author (yintaosun123@163.com).
