# Supplementary figures and images for: Elucidating the role of AC026412.3 in hepatocellular carcinoma: a prognostic disulfidptosis-related LncRNAs model perspective
Source: BMC Gastroenterol. 2025 Aug 12;25:579. doi: 10.1186/s12876-025-04174-6 (PMC12341353; doi:10.1186/s12876-025-04174-6)

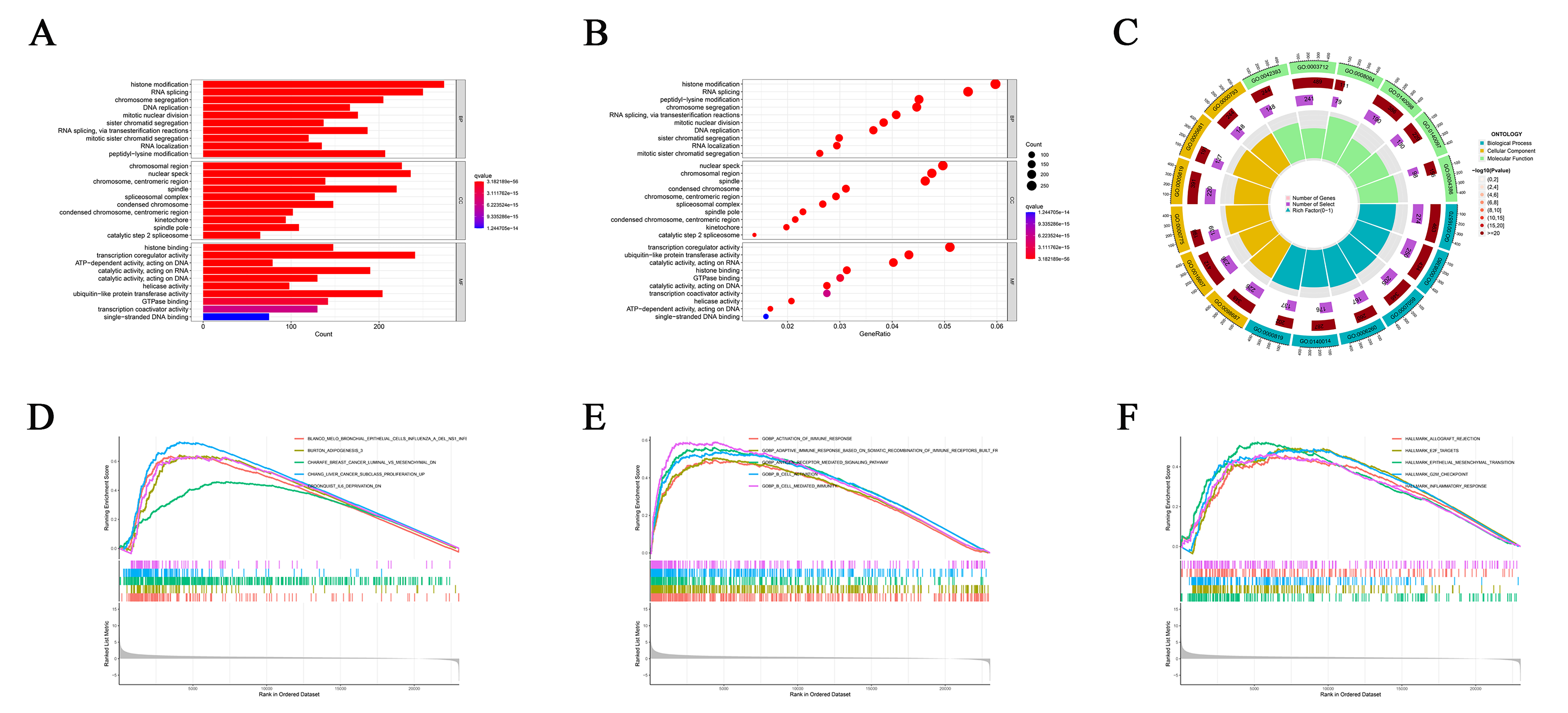

Supplement: Supplementary file 3 — Supplementary Figure 3. Gene Ontology (GO) and Gene Set Enrichment Analysis (GSEA) for AL031985.3. (A) Bar charts of the top 10 enriched Gene Ontology (GO) terms in the categories of Biological Processes (BP), Cellular Components (CC), and Molecular Functions (MF). The vertical axis represents the GO term names, while the horizontal axis denotes the number of genes enriched in each biological pathway. The color of the bars represents the significance of enrichment. (B) Bubble chart illustrating the top 10 enriched GO terms for BP, CC, and MF. The vertical axis represents the names of the GO terms, while the horizontal axis denotes the proportion of genes associated with each term. The size of the bubbles corresponds to the number of genes enriched in each GO term, and the color of the bubbles indicates the significance of the enrichment. (C) The enrichment circular chart represents the GO analysis of co-expressed genes associated with AL031985.3. The plot utilises three distinct colour codes: teal, tawny, and bright green, which correspond to BP, CC, and MF, respectively. The first ring displays the top six GO terms for each category. The second ring illustrates the number of genes in the genomic background and the P-values for gene enrichment associated with the specified GO terms, where the colour intensity reflects the P-value for enrichment. The third ring denotes the number of co-expressed genes enriched in the GO term. The fourth ring represents the enrichment factor for each GO term, indicating the proportion of genes. GSEA shows significant differences in enrichment in the TCGA HCC cohort for the c2.all.v2022.1.Hs.symbols.gmt gene set between the AL031985.3 high-expression group and low-expression group (D), and for the c5.all.v2022.1.Hs.symbols.gmt gene set between the AL031985.3 high-expression group and low-expression group (E). Significant enrichment in the h.all.v2022.1.Hs.symbols.cmt gene set was found in the AL031985.3 high-expression group (F). The x [file 12876_2025_4174_MOESM3_ESM.tif]

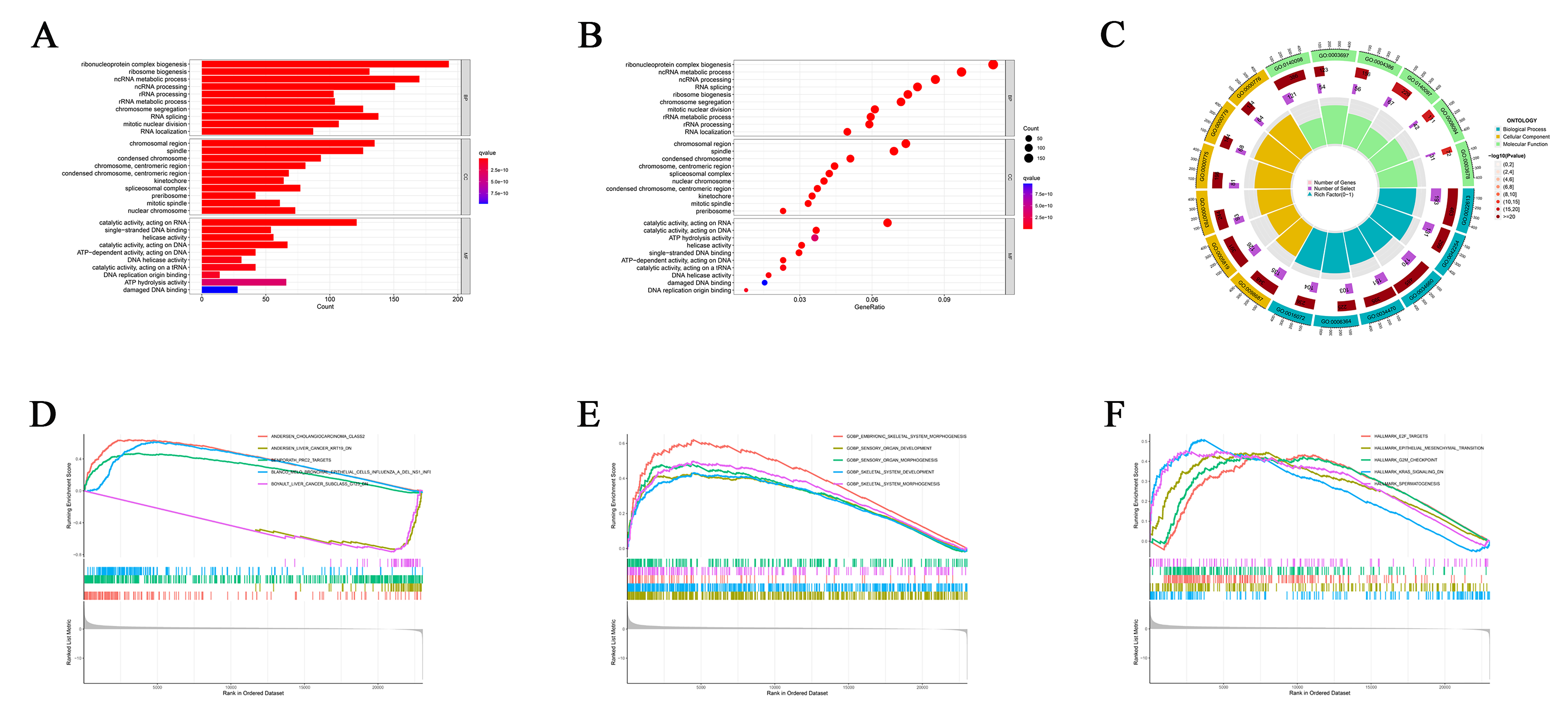

Supplement: Supplementary file 4 — Supplementary Figure 4. GO and GSEA for TMCC1-AS1. (A) Bar charts of the top 10 enriched GO terms in the categories of BP, CC, and MF. The vertical axis represents the GO term names, while the horizontal axis denotes the number of genes enriched in each biological pathway. The color of the bars represents the significance of enrichment. (B) Bubble chart illustrating the top 10 enriched GO terms for BP, CC, and MF. The vertical axis represents the names of the GO terms, while the horizontal axis denotes the proportion of genes associated with each term. The size of the bubbles corresponds to the number of genes enriched in each GO term, and the color of the bubbles indicates the significance of the enrichment. (C) The enrichment circular chart represents the GO analysis of co-expressed genes associated with TMCC1-AS1. The plot utilises three distinct colour codes: teal, tawny, and bright green, which correspond to BP, CC, and MF, respectively. The first ring displays the top six GO terms for each category. The second ring illustrates the number of genes in the genomic background and the P-values for gene enrichment associated with the specified GO terms, where the colour intensity reflects the P-value for enrichment. The third ring denotes the number of co-expressed genes enriched in the GO term. The fourth ring represents the enrichment factor for each GO term, indicating the proportion of genes. GSEA shows significant differences in enrichment in the TCGA HCC cohort for the c2.all.v2022.1.Hs.symbols.gmt gene set between the TMCC1-AS1 high-expression group and low-expression group (D), and for the c5.all.v2022.1.Hs.symbols.gmt gene set between the TMCC1-AS1 high-expression group and low-expression group (E). Significant enrichment in the h.all.v2022.1.Hs.symbols.cmt gene set was found in the TMCC1-AS1 high-expression group (F). The x-axis represents the ranked genes, while the y-axis indicates the enrichment scores. The curves in different colors correspond to disti [file 12876_2025_4174_MOESM4_ESM.tif]

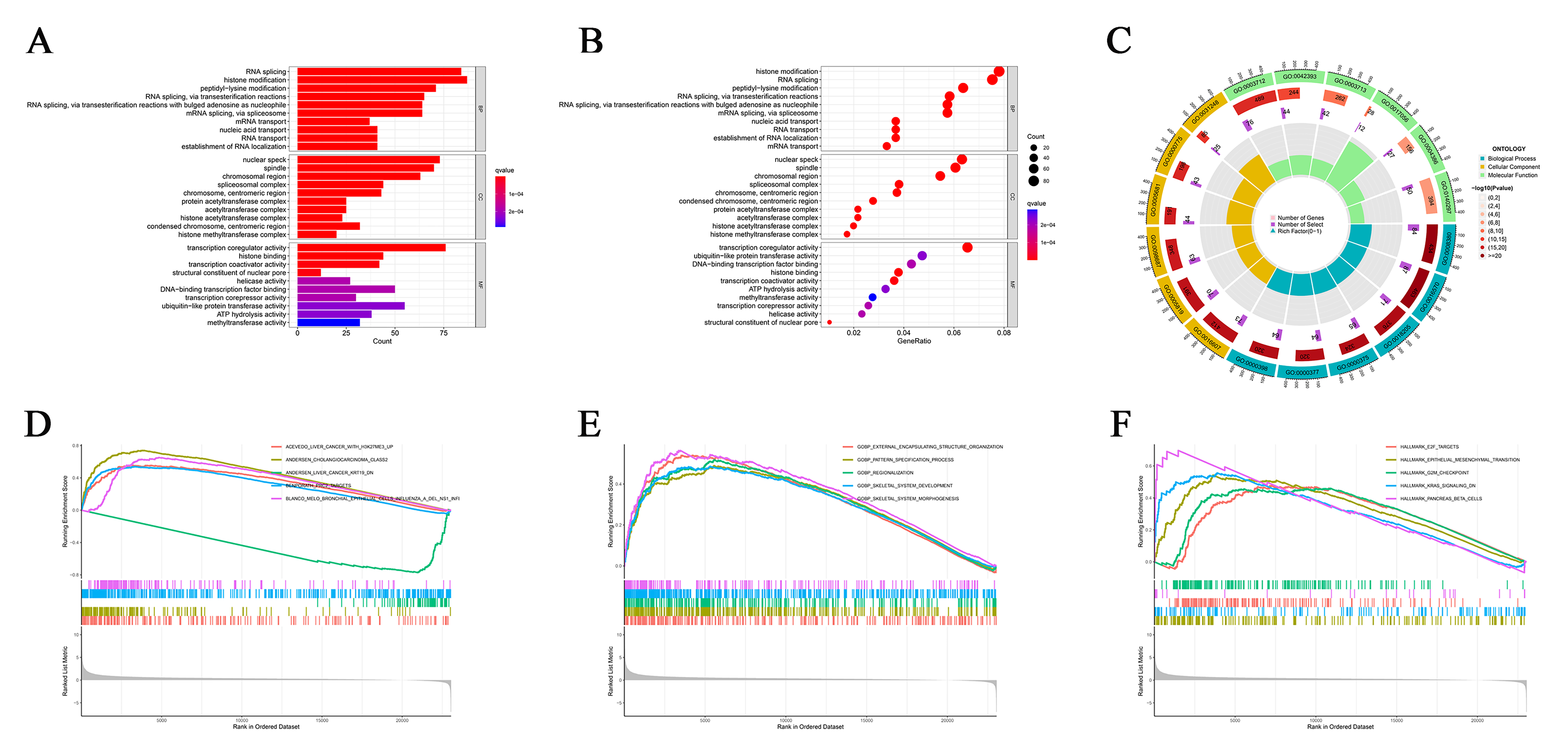

Supplement: Supplementary file 5 — Supplementary Figure 5. GO and GSEA for AL590705.3. (A) Bar charts of the top 10 enriched GO terms in the categories of BP, CC, and MF. The vertical axis represents the GO term names, while the horizontal axis denotes the number of genes enriched in each biological pathway. The color of the bars represents the significance of enrichment. (B) Bubble chart illustrating the top 10 enriched GO terms for BP, CC, and MF. The vertical axis represents the names of the GO terms, while the horizontal axis denotes the proportion of genes associated with each term. The size of the bubbles corresponds to the number of genes enriched in each GO term, and the color of the bubbles indicates the significance of the enrichment. (C) The enrichment circular chart represents the GO analysis of co-expressed genes associated with AL590705.3. The plot utilises three distinct colour codes: teal, tawny, and bright green, which correspond to BP, CC, and MF, respectively. The first ring displays the top six GO terms for each category. The second ring illustrates the number of genes in the genomic background and the P-values for gene enrichment associated with the specified GO terms, where the colour intensity reflects the P-value for enrichment. The third ring denotes the number of co-expressed genes enriched in the GO term. The fourth ring represents the enrichment factor for each GO term, indicating the proportion of genes. GSEA shows significant differences in enrichment in the TCGA HCC cohort for the c2.all.v2022.1.Hs.symbols.gmt gene set between the AL590705.3 high-expression group and low-expression group (D), and for the c5.all.v2022.1.Hs.symbols.gmt gene set between the AL590705.3 high-expression group and low-expression group (E). Significant enrichment in the h.all.v2022.1.Hs.symbols.cmt gene set was found in the AL590705.3 high-expression group (F). The x-axis represents the ranked genes, while the y-axis indicates the enrichment scores. The curves in different colors correspond to [file 12876_2025_4174_MOESM5_ESM.tif]

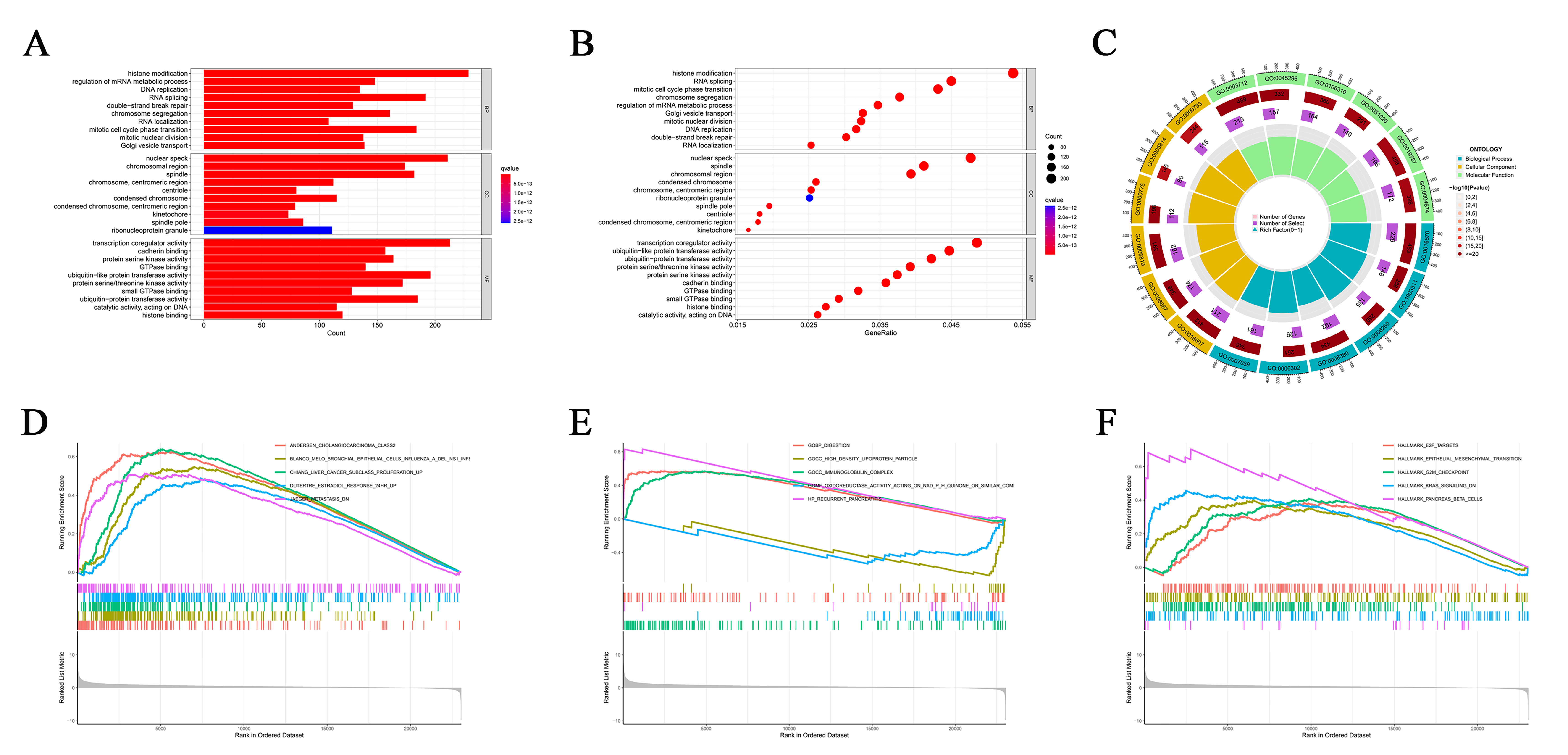

Supplement: Supplementary file 6 — Supplementary Figure 6. GO and GSEA for AC026412.3. (A) Bar charts of the top 10 enriched GO terms in the categories of BP, CC, and MF. The vertical axis represents the GO term names, while the horizontal axis denotes the number of genes enriched in each biological pathway. The color of the bars represents the significance of enrichment. (B) Bubble chart illustrating the top 10 enriched GO terms for BP, CC, and MF. The vertical axis represents the names of the GO terms, while the horizontal axis denotes the proportion of genes associated with each term. The size of the bubbles corresponds to the number of genes enriched in each GO term, and the color of the bubbles indicates the significance of the enrichment. (C) The enrichment circular chart represents the GO analysis of co-expressed genes associated with AC026412.3. The plot utilises three distinct colour codes: teal, tawny, and bright green, which correspond to BP, CC, and MF, respectively. The first ring displays the top six GO terms for each category. The second ring illustrates the number of genes in the genomic background and the P-values for gene enrichment associated with the specified GO terms, where the colour intensity reflects the P-value for enrichment. The third ring denotes the number of co-expressed genes enriched in the GO term. The fourth ring represents the enrichment factor for each GO term, indicating the proportion of genes. GSEA shows significant differences in enrichment in the TCGA HCC cohort for the c2.all.v2022.1.Hs.symbols.gmt gene set between the AC026412.3 high-expression group and low-expression group (D), and for the c5.all.v2022.1.Hs.symbols.gmt gene set between the AC026412.3 high-expression group and low-expression group (E). Significant enrichment in the h.all.v2022.1.Hs.symbols.cmt gene set was found in the AC026412.3 high-expression group (F). The x-axis represents the ranked genes, while the y-axis indicates the enrichment scores. The curves in different colors correspond to [file 12876_2025_4174_MOESM6_ESM.tif]

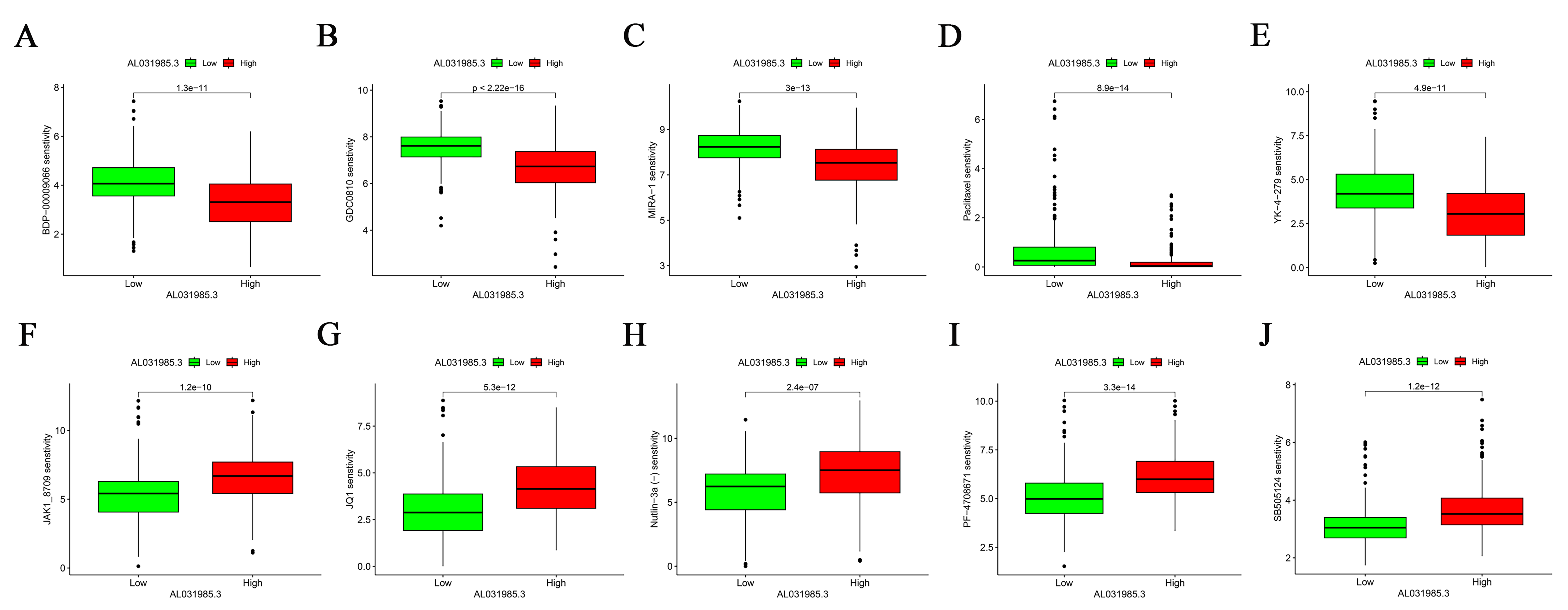

Supplement: Supplementary file 7 — Supplementary Figure 7. Drug sensitivity in HCC patients with high and low expression of AL031985.3. The x-axis represents groups with high and low expression levels of AL031985.3, while the y-axis indicates drug sensitivity. [file 12876_2025_4174_MOESM7_ESM.tif]

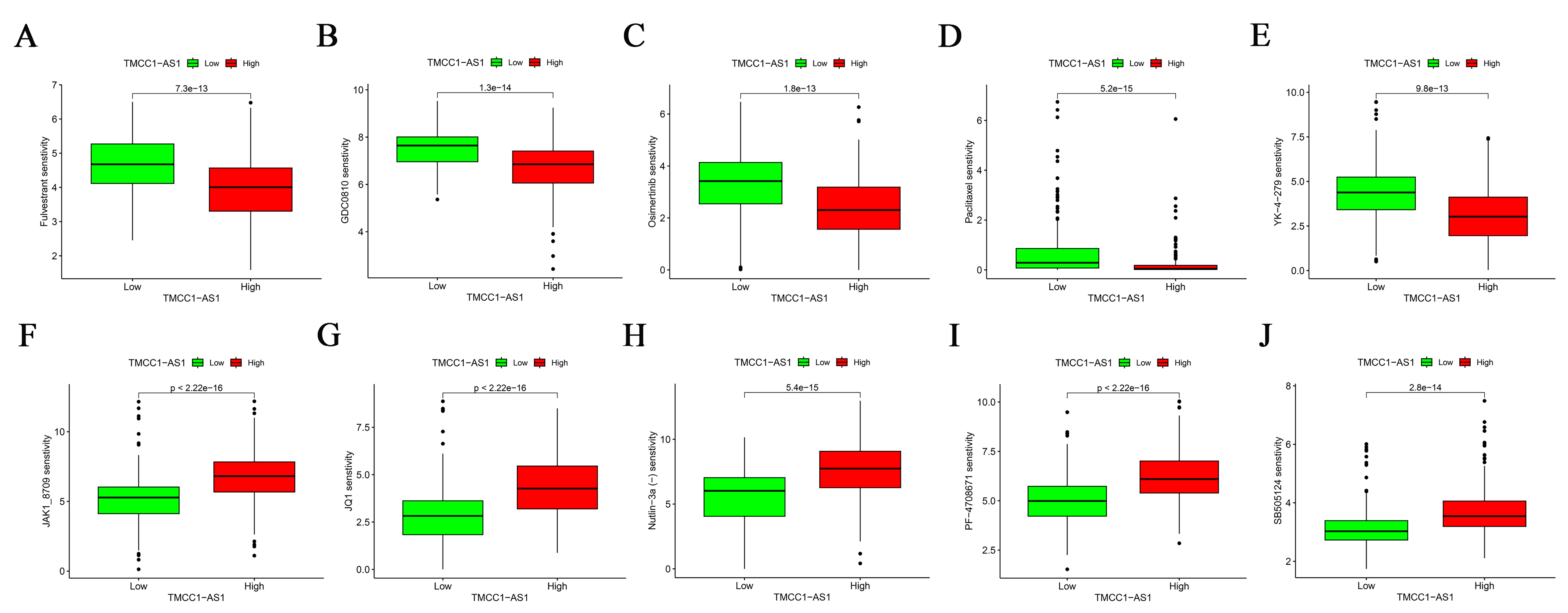

Supplement: Supplementary file 8 — Supplementary Figure 8. Drug sensitivity in HCC patients with high and low expression of TMCC1-AS1. The x-axis represents groups with high and low expression levels of TMCC1-AS1, while the y-axis indicates drug sensitivity. [file 12876_2025_4174_MOESM8_ESM.tif]

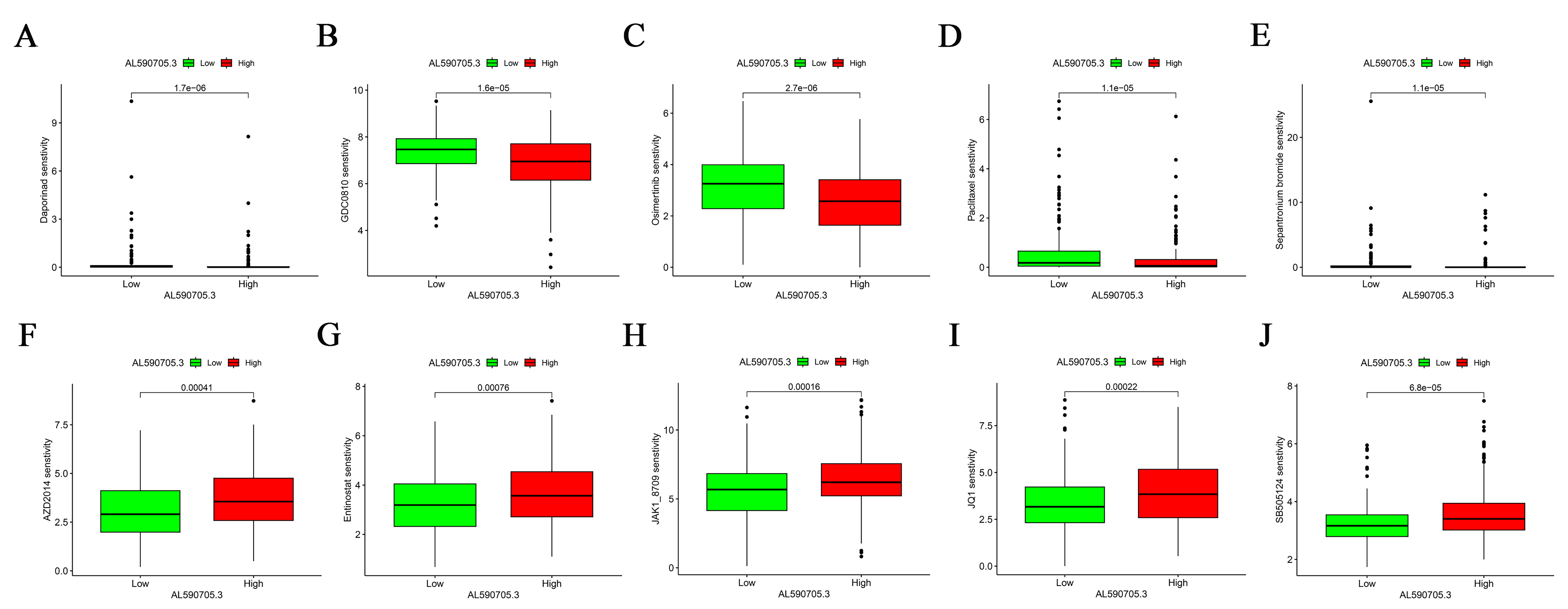

Supplement: Supplementary file 9 — Supplementary Figure 9. Drug sensitivity in HCC patients with high and low expression of AL590705.3. The x-axis represents groups with high and low expression levels of AL590705.3, while the y-axis indicates drug sensitivity. [file 12876_2025_4174_MOESM9_ESM.tif]

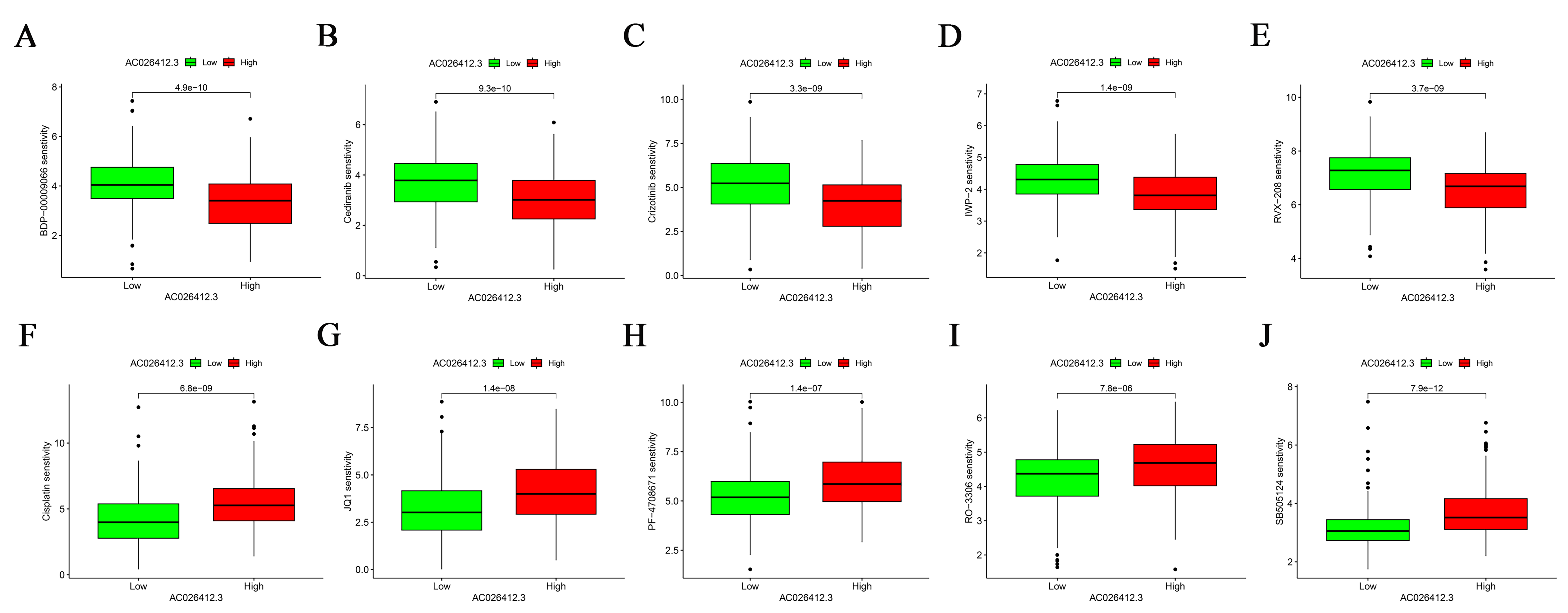

Supplement: Supplementary file 10 — Supplementary Figure 10. Drug sensitivity in HCC patients with high and low expression of AC026412.3. The x-axis represents groups with high and low expression levels of AC026412.3, while the y-axis indicates drug sensitivity. [file 12876_2025_4174_MOESM10_ESM.tif]

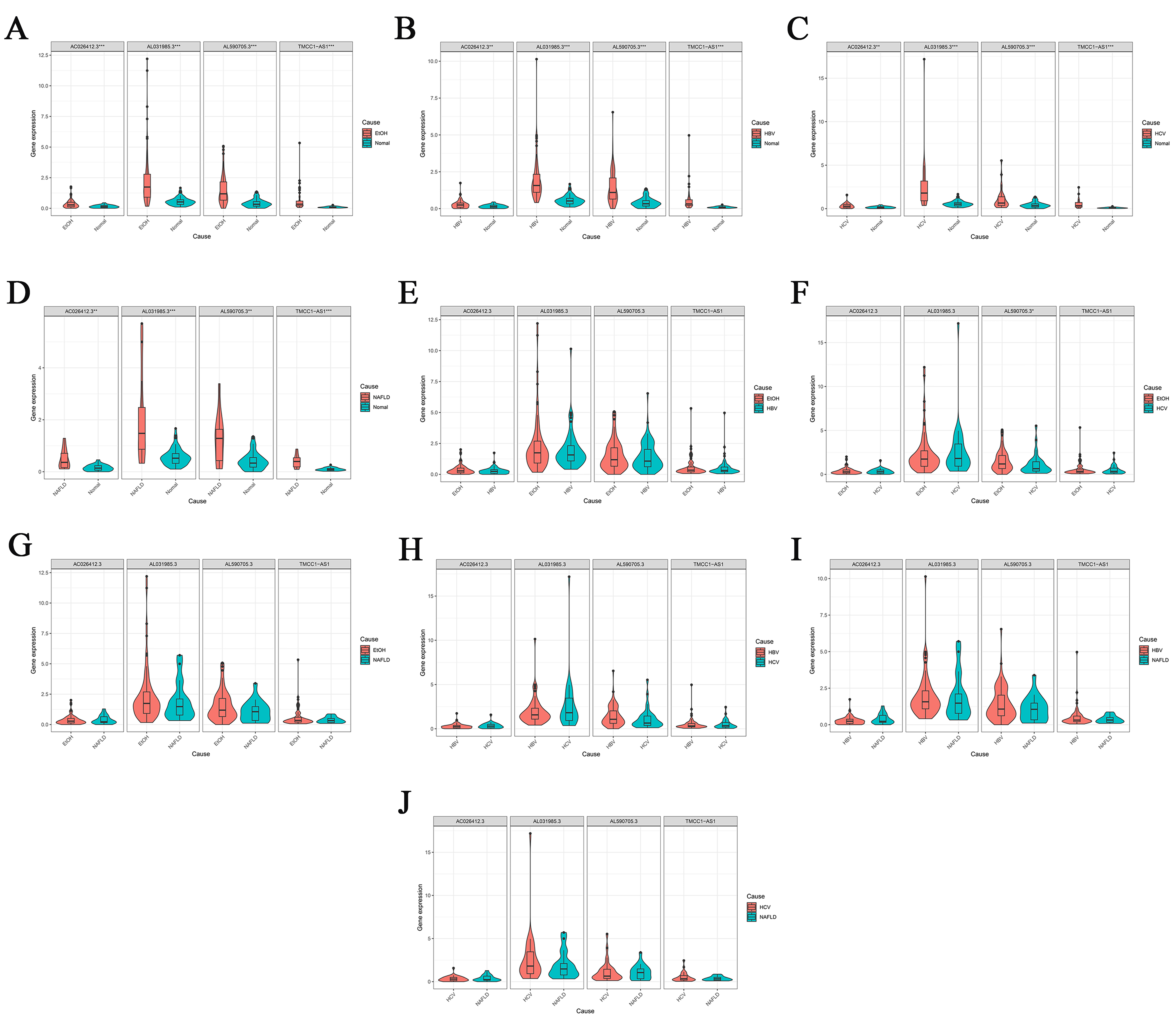

Supplement: Supplementary file 11 — Supplementary Figure 11. Expression patterns of four DRLs in HCC of different aetiologies based on data from The Cancer Genome Atlas (TCGA). (A) Comparative analysis of four DRLs expression between HCC tissues with alcohol consumption (EtOH) as the aetiological factor and adjacent normal tissues. (B) Comparative analysis of four DRLs expression in HCC tissues with hepatitis B as the aetiological factor versus adjacent normal tissues. (C) Comparative analysis of four DRLs expression in HCC tissues with hepatitis C as the aetiological factor versus adjacent normal tissues. (D) Comparative analysis of four DRLs expression in HCC tissues with Non-Alcoholic Fatty Liver Disease (NAFLD) as the aetiological factor versus adjacent normal tissues. (E) Comparative expression analysis of four DRLs between ethanol-induced and hepatitis B-induced HCC. (F) Comparative expression analysis of four DRLs between ethanol-induced and hepatitis C-induced HCC. (G) Comparative expression analysis of four DRLs between ethanol-induced and NAFLD-induced HCC. (H) Comparative expression analysis of four DRLs between hepatitis B-induced and hepatitis C-induced HCC. (I) Comparative expression analysis of four DRLs between hepatitis B-induced and NAFLD-induced HCC. (J) Comparative expression analysis of four DRLs between hepatitis C-induced and NAFLD-induced HCC. [file 12876_2025_4174_MOESM11_ESM.tif]
