# Supplementary material for: Elucidating the role of AC026412.3 in hepatocellular carcinoma: a prognostic disulfidptosis-related LncRNAs model perspective
Source: BMC Gastroenterol. 2025 Aug 12;25:579. doi: 10.1186/s12876-025-04174-6 (PMC12341353; doi:10.1186/s12876-025-04174-6)
Supplement: Supplementary file 12 — Supplementary Table 1. Features of hepatocellular carcinoma (HCC) patients in the complete cohort, training cohort, and validation cohort. [file 12876_2025_4174_MOESM12_ESM.docx]

**Supplementary Table 1:** Features of HCC patients in the complete cohort, training cohort, and validation cohort

| Covariates | Category | Total (n, % ) | Train (n, % ) | Test (n, % ) | Pvalue | EffectSize (95%CI) | EffectType |
| --- | --- | --- | --- | --- | --- | --- | --- |
| Age | <=65 | 227 (62.19%) | 117 (63.93%) | 110 (60.44%) | 0.5616 | 0.862 (0.552-1.346) | OR |
|  | >65 | 138 (37.81%) | 66 (36.07%) | 72 (39.56%) |  |  |  |
| Gender | FEMALE | 119 (32.6%) | 63 (34.43%) | 56 (30.77%) | 0.5264 | 0.847 (0.533-1.343) | OR |
|  | MALE | 246 (67.4%) | 120 (65.57%) | 126 (69.23%) |  |  |  |
| Grade | G1 | 55 (15.07%) | 30 (16.39%) | 25 (13.74%) | 0.5799 | 0.074 (0.003-0.125) | Cramer's V |
|  | G2 | 175 (47.95%) | 86 (46.99%) | 89 (48.9%) |  |  |  |
|  | G3 | 118 (32.33%) | 61 (33.33%) | 57 (31.32%) |  |  |  |
|  | G4 | 12 (3.29%) | 4 (2.19%) | 8 (4.4%) |  |  |  |
|  | unknow | 5 (1.37%) | 2 (1.09%) | 3 (1.65%) |  |  |  |
| Stage | Stage I | 170 (46.58%) | 87 (47.54%) | 83 (45.6%) | 0.7788 | 0.057 (0.01-0.088) | Cramer's V |
|  | Stage II | 84 (23.01%) | 38 (20.77%) | 46 (25.27%) |  |  |  |
|  | Stage III | 83 (22.74%) | 38 (20.77%) | 45 (24.73%) |  |  |  |
|  | Stage IV | 4 (1.1%) | 2 (1.09%) | 2 (1.1%) |  |  |  |
|  | unknow | 24 (6.58%) | 18 (9.84%) | 6 (3.3%) |  |  |  |
| T | T1 | 180 (49.32%) | 93 (50.82%) | 87 (47.8%) | 0.4247 | 0.103 (0.057-0.142) | Cramer's V |
|  | T2 | 91 (24.93%) | 42 (22.95%) | 49 (26.92%) |  |  |  |
|  | T3 | 78 (21.37%) | 37 (20.22%) | 41 (22.53%) |  |  |  |
|  | T4 | 13 (3.56%) | 9 (4.92%) | 4 (2.2%) |  |  |  |
|  | TX | 1 (0.27%) | 1 (0.55%) | 0 (0%) |  |  |  |
|  | unknow | 2 (0.55%) | 1 (0.55%) | 1 (0.55%) |  |  |  |
| M | M0 | 263 (72.05%) | 130 (71.04%) | 133 (73.08%) | 0.7962 | 0.035 (0.001-0.062) | Cramer's V |
|  | M1 | 3 (0.82%) | 2 (1.09%) | 1 (0.55%) |  |  |  |
|  | MX | 99 (27.12%) | 51 (27.87%) | 48 (26.37%) |  |  |  |
| N | N0 | 248 (67.95%) | 124 (67.76%) | 124 (68.13%) | 0.5958 | 0.053 (0.001-0.099) | Cramer's V |
|  | N1 | 4 (1.1%) | 1 (0.55%) | 3 (1.65%) |  |  |  |
|  | NX | 112 (30.68%) | 57 (31.15%) | 55 (30.22%) |  |  |  |
|  | unknow | 1 (0.27%) | 1 (0.55%) | 0 (0%) |  |  |  |

**Abbreviations:** CI: confidence interval, Cramer's V: Cramer's V coefficient, HCC: hepatocellular carcinoma, OR: Odds Ratio, TNM: tumor node metastasis.
