# Supplementary material for: Elucidating the role of AC026412.3 in hepatocellular carcinoma: a prognostic disulfidptosis-related LncRNAs model perspective
Source: BMC Gastroenterol. 2025 Aug 12;25:579. doi: 10.1186/s12876-025-04174-6 (PMC12341353; doi:10.1186/s12876-025-04174-6)
Supplement: Supplementary file 13 — Supplementary Table 2. Clinical characteristics and median risk score distribution across treatment groups. [file 12876_2025_4174_MOESM13_ESM.docx]

**Supplementary Table 2:** Clinical characteristics and median risk score distribution across treatment groups

| Treatment Group | n | median_age | male (%) | female (%) | median_risk |
| --- | --- | --- | --- | --- | --- |
| Surgery Only | 311 | 61.000 | 211 (67.8%) | 100 (32.2%) | 0.773 |
| Adjuvant Therapy | 16 | 64.500 | 8 (50.0%) | 8 (50.0%) | 1.100 |
| Ablation/Embolization | 12 | 59.500 | 9 (75.0%) | 3 (25.0%) | 0.613 |
| Other/Unknown | 26 | 62.500 | 18 (69.2%) | 8 (30.8%) | 1.029 |
