# Supplementary material for: Elucidating the role of AC026412.3 in hepatocellular carcinoma: a prognostic disulfidptosis-related LncRNAs model perspective
Source: BMC Gastroenterol. 2025 Aug 12;25:579. doi: 10.1186/s12876-025-04174-6 (PMC12341353; doi:10.1186/s12876-025-04174-6)
Supplement: Supplementary file 14 — Supplementary Table 3. Cox proportional hazards model with interaction terms. [file 12876_2025_4174_MOESM14_ESM.docx]

**Supplementary Table 3:** Cox proportional hazards model with interaction terms

| Variable | coef | exp(coef) | se(coef) | z | Pr(>\|z\|) |
| --- | --- | --- | --- | --- | --- |
| riskScore | 0.039 | 1.040 | 0.014 | 2.706 | 0.007 |
| treatment_group Adjuvant Therapy | 0.836 | 2.306 | 0.717 | 1.165 | 0.244 |
| treatment_group Ablation/Embolization | -1.010 | 0.364 | 0.871 | -1.159 | 0.246 |
| treatment_group Other/Unknown | 0.203 | 1.225 | 0.419 | 0.484 | 0.628 |
| age | 0.017 | 1.017 | 0.008 | 2.274 | 0.023 |
| Gender Male | -0.104 | 0.901 | 0.194 | -0.535 | 0.592 |
| riskScore:treatment_group Adjuvant Therapy | -0.330 | 0.719 | 0.474 | -0.696 | 0.487 |
| riskScore:treatment_group Ablation/Embolization | 0.944 | 2.570 | 0.632 | 1.493 | 0.135 |
| riskScore:treatment_group Other/Unknown | 0.251 | 1.285 | 0.062 | 4.037 | 0.000 |

**Abbreviations:** coef: Coefficient, exp(coef): Exponentiated Coefficient, Pr(>|z|) : P-value of z-statistic, se(coef): Standard Error of Coefficient, z: z-statistic.
