# Supplementary material for: Elucidating the role of AC026412.3 in hepatocellular carcinoma: a prognostic disulfidptosis-related LncRNAs model perspective
Source: BMC Gastroenterol. 2025 Aug 12;25:579. doi: 10.1186/s12876-025-04174-6 (PMC12341353; doi:10.1186/s12876-025-04174-6)
Supplement: Supplementary file 15 — Supplementary Material 15. [file 12876_2025_4174_MOESM15_ESM.zip › Supplementary methods section/Supplementary_Methods_Protocol.docx]

**Supplementary Methods Protocol**

**Prognostic Model Construction**

**Version:** 1.0
**Date:** June 18, 2025

**Table of Contents**

1. Software Environment
2. Step-by-Step Protocol
   - 2.1 Data Preprocessing
   - 2.2 Data Partitioning
   - 2.3 Feature Selection
   - 2.4 Model Construction
   - 2.5 Model Validation
3. Output Files
4. Reproducibility Notes

**1. Software Environment**

**1.1 Core Software**

- **R version:** 4.3.1 (2023-6-16)
- **Operating System:** Platform independent (Windows/Linux/macOS)

**1.2 R Packages**

| **Package** | **Version** | **Purpose** |
| --- | --- | --- |
| survival | 3.8-3 | Survival analysis |
| caret | 7.0-1 | Data splitting |
| glmnet | 4.1-8 | LASSO regression |
| timeROC | 0.4 | Time-dependent ROC analysis |
| survminer | 0.5.0 | Survival visualization |

**1.3 Perl Script**

- **File:** biotype.pl
- **Purpose:** Separate lncRNA and mRNA from RNA-seq
- **Input:** Raw sequencing data
- **Output:** mRNA.txt: mRNA expression matrix (from biotype.pl);lncRNA.txt: lncRNA expression matrix (from biotype.pl)

**2. Step-by-Step Protocol**

**2.1 Data Preprocessing**

**Input Requirements**

- **File format:** Tab-delimited text file (expTime.txt)
- **Columns:**
  - futime: Survival time in days
  - fustat: Event status (0 = censored, 1 = event)
  - Subsequent columns: Gene expression values

**Processing Steps:**

1. **Survival time adjustment:**
   - Convert days to years: futime/365
   - Replace non-positive values: futime[futime <= 0] = 1
2. **Expression transformation:**
   - Apply log2 normalization: log2(expression + 1)
3. **Data structure:**
   - Rows: Samples
   - Columns: Clinical variables + genes

**2.2 Data Partitioning**

- **Method:** Stratified random sampling
- **Partition ratio:** 50% training / 50% testing
- **Stratification:** Based on event status (fustat)
- **R function:** createDataPartition() from caret package
- **Seed:** Set to 123 for reproducibility

**2.3 Feature Selection**

**Step 1: Univariate Cox Regression**

- **Inclusion criteria:**
  - Standard deviation > 0.1
  - Cox P-value < 0.05
- **Output files:**
  - uni.trainCox.txt: Significant genes with HR, 95% CI, P-value
  - uni.SigExp.txt: Expression matrix of significant genes
- **Visualization:** Forest plot (uni.forest.pdf)

**Step 2: LASSO Cox Regression**

- **Configuration parameters:**
  - Family: cox (proportional hazards)
  - Alpha: 1 (L1 penalty)
  - maxit: 1000 (maximum iterations)
  - Convergence tolerance: 1e-5
- **Cross-validation:**
  - Method: 10-fold CV
  - Metric: Partial likelihood deviance
  - Lambda selection: lambda.min (minimizes cross-validated error)
- **Output files:**
  - lasso.lambda.pdf: Coefficient paths
  - lasso.cvfit.pdf: Cross-validation error
  - lasso.SigExp.txt: LASSO-selected genes

**2.4 Model Construction**

**Multivariate Cox Regression**

- **Input:** LASSO-selected genes
- **Variable selection:**
  - Method: Stepwise regression
  - Direction: Both (forward-backward)
  - Criterion: Akaike Information Criterion (AIC)
  - Threshold: Î”AIC < 2.0
- **Risk score calculation**
